# Sustained-release lidocaine sheet for pain following tooth extraction: A randomized, single-blind, dose-response, controlled, clinical study of efficacy and safety

**DOI:** 10.1371/journal.pone.0200059

**Published:** 2018-07-02

**Authors:** Toshiyuki Suzuki, Kensuke Kosugi, Takashi Suto, Masaru Tobe, Yasuhiko Tabata, Satoshi Yokoo, Shigeru Saito

**Affiliations:** 1 Department of Anesthesiology, Gunma University Graduate School of Medicine, Maebashi-shi, Gunma, Japan; 2 Department of Oral and Maxillofacial Surgery, Gunma University Graduate School of Medicine, Maebashi-shi, Gunma, Japan; 3 Department of Biomaterials, Field of Tissue Engineering, Institute for Frontier Medical Sciences, Kyoto University, Kyoto-shi, Kyoto, Japan; Tokai Daigaku, JAPAN

## Abstract

**Background:**

We have synthesized a sustained-release lidocaine sheet (SRLS) using biodegradable polymers and previously demonstrated its safety and long-term analgesic effect in the normal mucous membrane of healthy human volunteers.

**Objectives:**

The aim of this clinical study was to evaluate the efficacy, safety, and appropriate dose of the SRLS for pain following tooth extraction.

**Design:**

Randomized, single-blind, dose-response, controlled, clinical study (Phase 1/2).

**Methods:**

The patients in this trial were enrolled between January 2014 and December 2016. A total of 99 patients were randomly divided into 5 groups as follows: the Non-administration group received the conventional extraction; the Poly Lactic-co-Glycolic Acid (PLGA) 100 mg control group received the PLGA matrix without lidocaine; the SRLS 100 mg group received a single sheet of SRLS 100 mg; the SRLS 200 mg group received double sheets of SRLS 100 mg; and the SRLS 400 mg administration group received four sheets of SRLS 100 mg. A study drug was inserted into the defect socket after the extraction, and postoperative pain intensity, satisfaction with postoperative pain relief, adverse events, and postoperative supplemental analgesic rescue use (time, dose) were investigated by patient self-report.

**Results:**

In total, 94 (94.9%) patients completed the study. There were no significant differences in postoperative pain intensity, satisfaction with postoperative pain relief, and postoperative supplemental analgesic rescue use among the 5 groups. There were no serious side effects, including a plasma concentration increase of lidocaine, attributable to the SRLS.

**Conclusions:**

Administration of the SRLS at 100 mg may have clinical therapeutic potential for pain relief following tooth extraction. The safety of the SRLS for patients undergoing tooth extraction was demonstrated.

**Trial registration:**

The University Hospital Medical Information Network UMIN000011945

## Introduction

Postsurgical analgesia is a subject that has recently attracted growing interest, and various postsurgical analgesia methods based on a range of analgesics are used depending on such factors as the nature of the operation and the risk to the patient. However, the current situation in the field of postsurgical analgesia cannot be described as completely satisfactory. For example, operations on patients taking oral anticoagulants and anti-platelet agents have increased, and cases of early postoperative initiation of anticoagulant therapy have also increased. Accordingly, epidural anesthesia tends to be less frequently used due to the risk of hematoma-induced neuropathy. Continuous intravenous opioid infusion is one alternative that is being increasingly used [1]; however, opioids have a weak effect on movement-related pain, and they are associated with adverse reactions that prevent full pain relief, such as nausea, vomiting, drowsiness, and respiratory depression. Ultrasound-guided peripheral nerve blocks have also recently been increasingly used as the resolution of the equipment improves. Such nerve blocks represent a safe and very satisfactory method of pain relief [2–4]; however, the necessity for technology acquisition and the substantial early-phase costs are problematic.

Long-acting analgesia requires continuous medication, whichever strategy is selected. Against this background, another approach involves the release of local anesthetics in small amounts over a prolonged period (sustained release). When achievable, it is considered that such an approach will be effective even for movement-related pain and will provide long-acting postsurgical analgesia through a single-injection at a surgical site, around a peripheral nerve, or into the epidural space without a continuous infusion catheter.

Postsurgical pain usually lasts several days. However, there is currently no U.S. Food and Drug Administration (FDA)-approved medication that reliably extends the duration of single-injection epidural or peripheral nerve blocks for over 24 hours.

In 2011, the FDA approved liposomal bupivacaine injectable suspension (EXPAREL^®^, Pacira Pharmaceuticals, Inc., Parsippany, NJ, USA) for infiltration into a surgical site to provide postsurgical analgesia in adults, based on clinical trials in subjects undergoing bunionectomy [5] and hemorrhoidectomy [6], which demonstrated efficacy for 36 h and 72 h, respectively. Furthermore, clinical trials of epidural block [7], femoral nerve block [8], transversus abdominis plane block [9,10] and interscalene block [11,12] with liposomal bupivacaine have been previously reported. The FDA has not yet approved liposomal bupivacaine for epidural or peripheral nerve blocks.

A sustained-release lidocaine sheet (SRLS) was developed using biodegradable material. The material, which is degraded and absorbed in the body and ultimately broken down into carbon dioxide and water, is already in clinical use as an absorbable suture. The SRLS is formulated differently from the liposomal bupivacaine, and its clinical use for patients has not been the subject of any international reports. We previously demonstrated the safety and efficacy of the SRLS for sciatic nerve block in a rat model of postoperative pain [13]. Furthermore, we have also synthesized injectable sustained-release lidocaine particles (SRLPs) that are not sheets from the same biodegradable polymers used for the SRLS, and we demonstrated that epidural injection of these particles produced a prolonged anti-hypersensitivity effect in a rat model of postoperative pain with no major complications [14]. Next, we demonstrated the duration and intensity of analgesia and the safety of the SRLS in the normal mucous membrane of healthy volunteers as an exploratory clinical trial prior to administration of the SRLS to patients [15]. The present study is the first clinical trial of the SRLS in patients.

## Materials and methods

This randomized, single-blind, dose-response, controlled, clinical study was approved by the Institutional Review Board of Gunma University Hospital (No.1079) and registered with the University Hospital Medical Information Network (UMIN000011945). Written, informed consent was obtained from all patients before enrollment. The patients were covered by liability insurance to support them in the event of unforeseen problems (physical disability, aftereffects, death, etc.) attributable to the SRLS. The aim of this clinical study was to evaluate the efficacy, safety, and appropriate dose of a sustained-release lidocaine sheet (SRLS) for pain following tooth extraction (Phase 1/2 clinical study).

### Subjects

**Fig 1:**
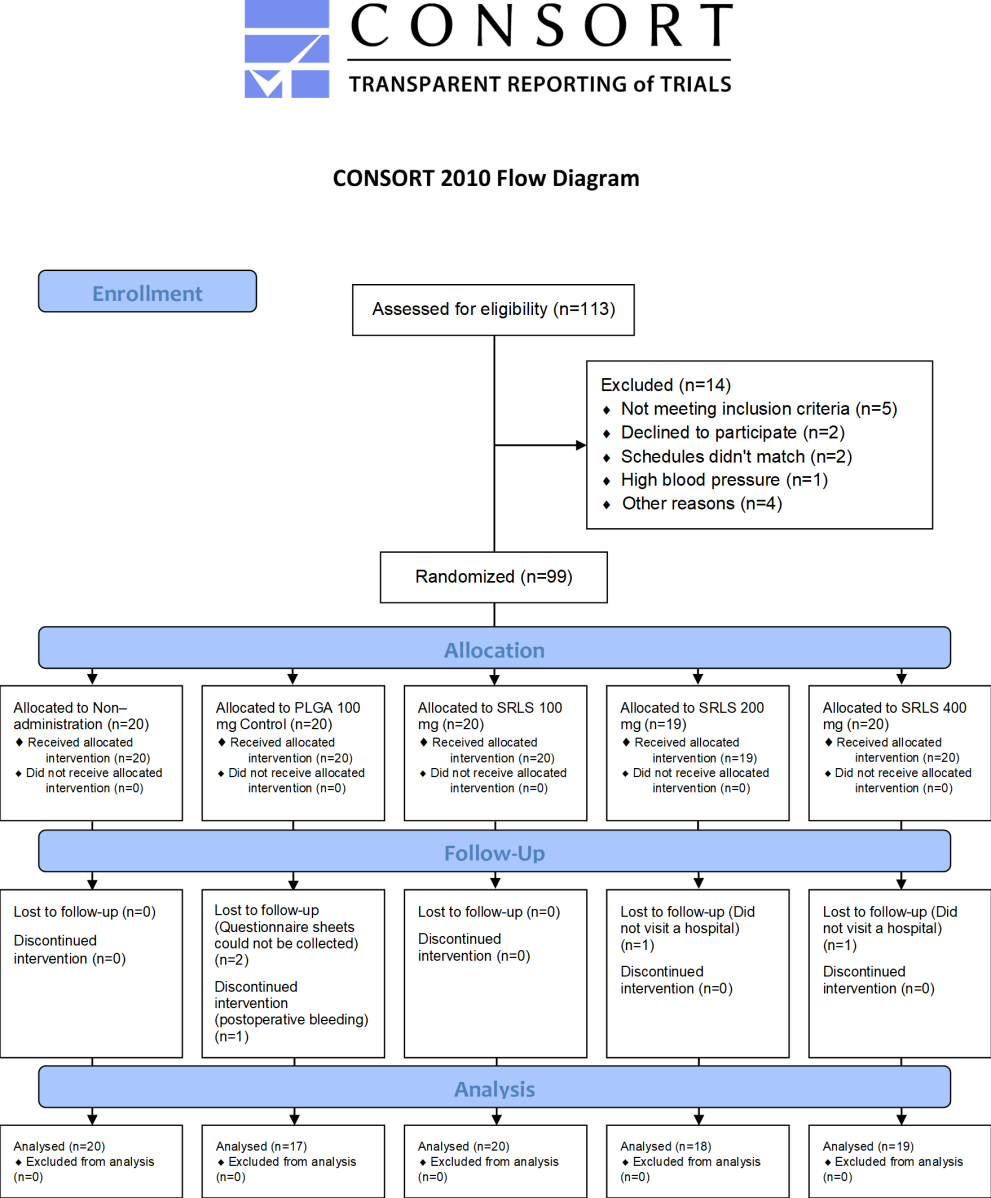
Patient disposition (CONSORT 2010 flow diagram). A total of 113 patients were enrolled, and 99 were randomized. Of the 99 patients randomized, 94 (94.9%) completed the study and were analyzed as the efficacy assessment population. The patient with postoperative bleeding and the 94 patients who completed the study, 95 patients in all, were analyzed as the safety assessment population.

The patients in this trial were enrolled from among outpatients of the Oral and Maxillofacial Surgery Department at Gunma University Hospital (Fig 1). They were screened prior to enrollment by interview regarding their medical history, and they underwent a blood examination to determine the hemoglobin concentration and red blood cell, white blood cell, and platelet counts. The blood samples were also subjected to biochemical examinations for total protein (TP), albumin, total bilirubin (T-bil), aspartate aminotransferase (AST), alanine aminotransferase (ALT), lactic acid dehydrogenase (LDH), alkaline phosphatase (ALP), γ-glutamyl transpeptidase (γ-GTP), cholinesterase, creatinine kinase (CK), amylase, blood urea nitrogen (BUN), creatinine, Na, K, Cl, Ca, glucose, and lidocaine concentration levels. The patients also underwent resting 12-lead electrocardiography (ECG).

Eligible patients were American Society of Anesthesiologists (ASA)—Physical Status (PS) 1 or 2 male and female outpatients aged 20 to 50 years, scheduled to undergo third molar extraction of a mandibular horizontally impacted wisdom tooth. Patients were not eligible if they took a medication considered to contribute to an analgesic effect (e.g. antipsychotics, non-steroidal anti-inflammatory drugs (NSAIDs), or opioids) or if a carry-over effect was considered possible if it had been discontinued, had a marked surgical site infection, peptic ulcer, asthma, allergy to lidocaine or amide type local anesthetics, celecoxib, sulfonamide, or acetaminophen, a serious disturbance of the heart conduction system (e.g. complete atrioventricular block), disturbance of consciousness or communication that would interfere with correct evaluation, or were judged unsuitable for this study by the attending doctor.

The patients were randomly divided into 5 groups: the non-administration group received the conventional extraction; the poly lactic-co-glycolic acid (PLGA) 100 mg control group received PLGA matrix without lidocaine; the SRLS 100 mg group received a single sheet of SRLS 100 mg; the SRLS 200 mg group received double sheets of SRLS 100 mg; and the SRLS 400 mg group received four sheets of SRLS 100 mg. Each group included 20 cases. Randomization was carried out using colored balls. A total of 50 balls (10 each of 5 different colors) were put into a box. To randomly allocate the cases, balls were drawn one by one, and the colors were reported to the operator. Balls that had been drawn once were not returned to the box until randomization was complete. After assigning 50 cases, the balls were returned to the box and randomization was repeated twice.

Based on our previous study [15], it was determined that a sample size of approximately 15 patients in each group would have 80% power to detect a difference of 104 and a standard deviation of 132 in the mean cumulative pain threshold using a *t*-test with a two-sided significance level of 0.05. Thus, a sample size of 20 patients in each group was planned to include missing values and drop-outs.

### Procedures

**Fig 2:**
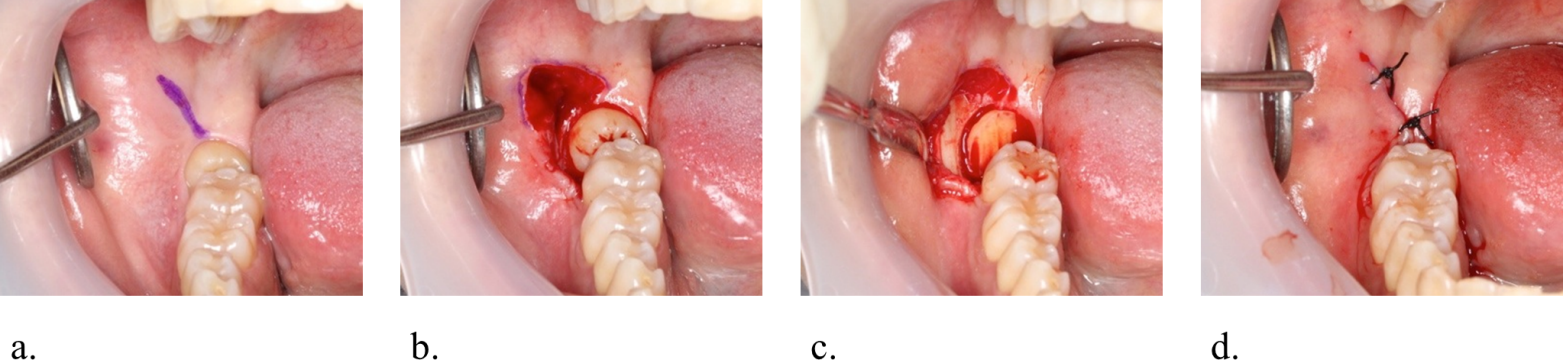
Exodontia procedure. a. Incision design for the third molar extraction. The envelope flap design without a vertical releasing incision, with lateral midcrestal incision to protect against lingual nerve damage. b. Bone resection before extraction of impacted wisdom teeth. The mandibular bone around the impacted wisdom teeth is removed with a surgical hand piece and burs to expose the cervical contour of the tooth. c. Crown sectioning with a surgical hand piece and burs. Extraction of the impacted portion usually requires crown sectioning, which prevents damage to the periodontium of the second molar and mandibular alveolar and lingual nerves. d. Wound closure following extraction of the tooth. The alveolar buccal margin is removed, and the defect socket is irrigated. Finally, the operator inserts a study drug into the socket and closes the incision by rough sutures to allow drainage.

**Fig 3:**
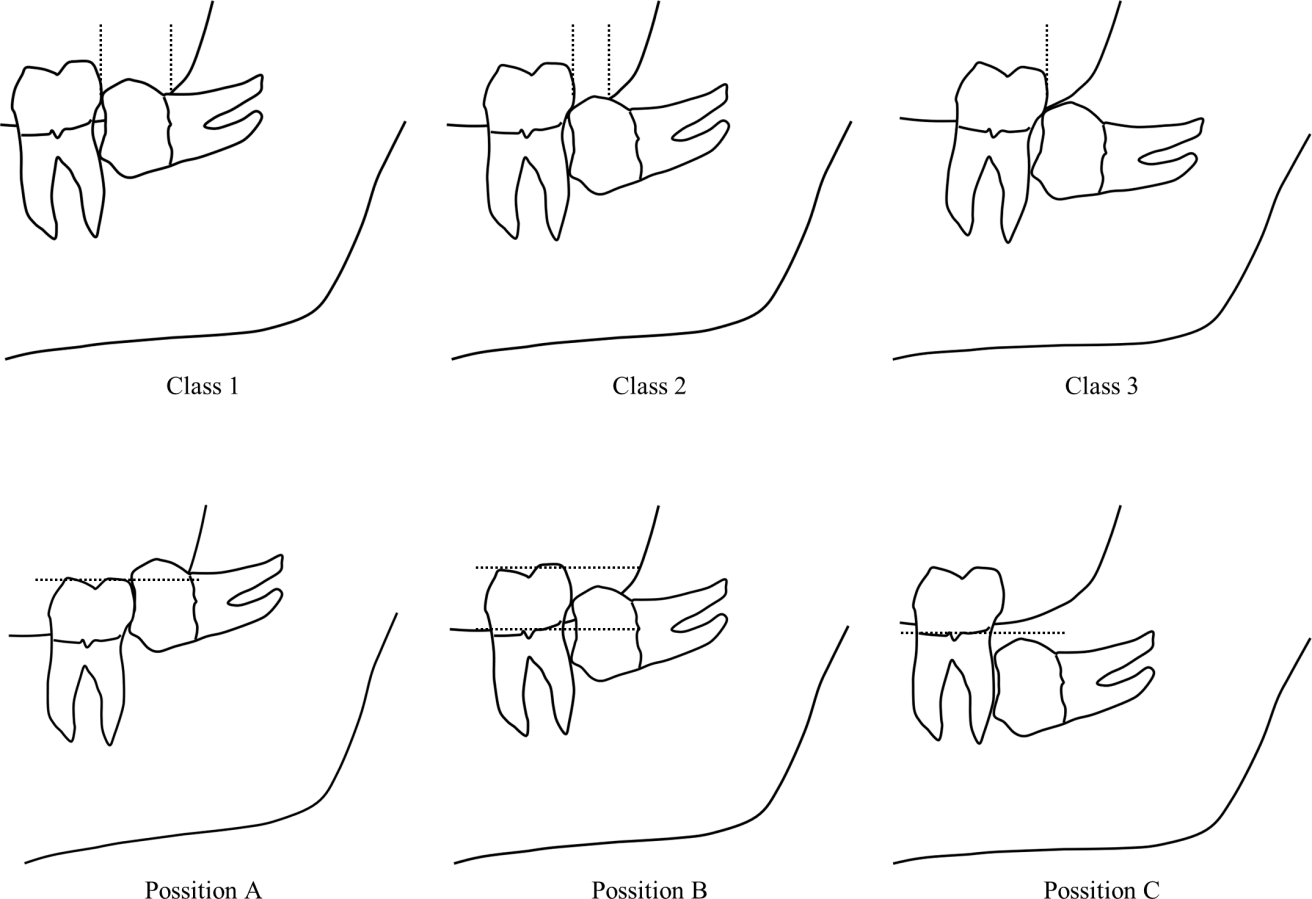
Pell & Gregory's classification. Class 1. There is sufficient space available between the anterior border of the ascending ramus and the distal aspect of the 2nd molar. The space is more than the mesio-distal width of the crown of the 3rd molar. Class 2. The space available between the anterior border of the ramus and the distal aspect of the 2nd molar is less than the mesio-distal width of the crown of the 3rd molar. It denotes that the distal portion of the 3rd molar crown is covered by bone of the ascending ramus. Class 3. The 3rd molar is totally embedded in the bone of the ascending ramus because of the absolute lack of the space. It is obvious that class 3 teeth present more difficulty in removal, since a relatively large amount of bone must be removed, and there is a risk of damaging the inferior dental (alveolar) nerve or fracturing the mandible, or both. Position A. The occlusal plane of the impacted tooth is at the same level as the occlusal plane of the 2nd molar, or above. (The highest portion of the impacted 3rd molar is on a level with the occlusal plane, or above). Position B. The occlusal plane of the impacted tooth is between the occlusal plane and the cervical margin of the 2nd molar. (The highest portion of the impacted 3rd molar is below the occlusal plane but above the cervical line of the 2nd molar). Position C. The occlusal plane of the impacted tooth is below the cervical margin of the 2nd molar. (The highest portion of the impacted 3rd molar is below the cervical line of the 2nd molar).

A dentist extracted a tooth in the classical manner using standard surgical tools (scalpel, retractor, forceps for tissues, needle holder, rongeur) and a surgical hand piece with high speed burs designed for odontotomy after local anesthesia with propitocaine hydrochloride and felypressin (a single cartridge contained 1.8 ml with propitocaine hydrochloride 54 mg and felypressin 0.054 units) added appropriately during the operation (Fig 2). The operator performed bone resection and odontotomy to avoid damaging the periodontium of the second molars and the mandibular alveolar and lingual nerves. The operator then inserted a study drug into the defect socket after the extraction and closed the surgical incision by sutures. The operator did not inform the patients about their assigned group. The remaining study drug was eliminated at the time of suture removal. All maneuvers of tooth extraction were performed by the same surgeon, who recorded medical information including height, weight, age, sex, and American Society of Anesthesiologists physical status classification (ASA-PS) of the patient, as well as location (right/left), dose of local anesthetics, operating time, the number of sutures, and Pell & Gregory’s classification (Fig 3). This classification is based on the amount of tooth covered by the anterior border of the ramus (Class 1, 2, or 3) and the depth of the impaction relative to the adjacent tooth (Position A, B, or C).

**Fig 4:**
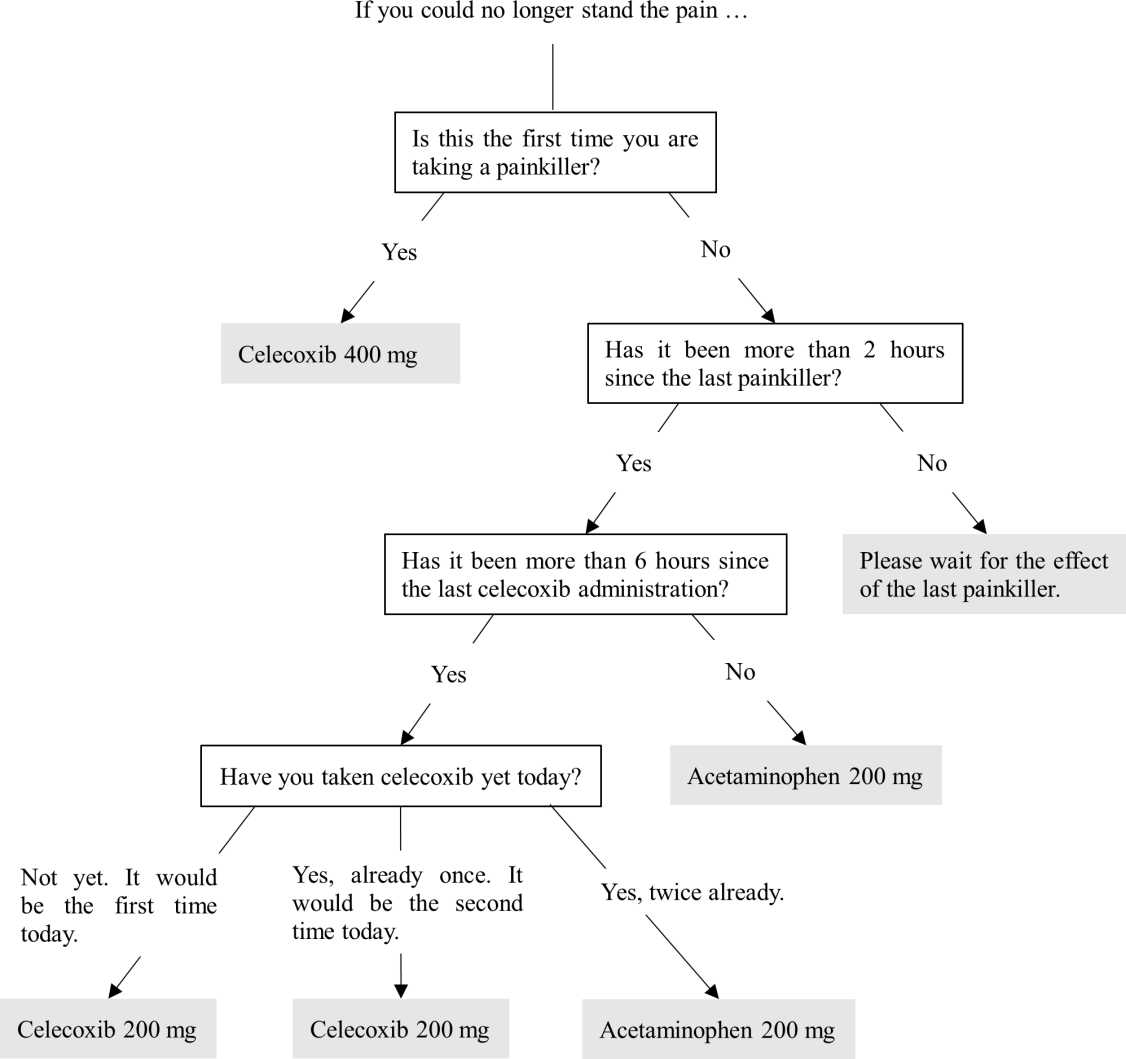
Analgesic flowchart. Patients could take celecoxib 200 mg (400 mg the first time) and repeat it a maximum of twice a day as needed. Furthermore, they could take acetaminophen 200 mg, both when more than two hours had passed after taking the last medication and when they could not wait until the next celecoxib. They could repeat acetaminophen to a maximum of 4,000 mg (20 times) a day as needed. However, when more than six hours had passed from the last administration of celecoxib, they took celecoxib prior to acetaminophen.

When patients needed analgesics, they could take an oral analgesic as rescue medication postoperatively according to the flow chart (Fig 4). The patients could take celecoxib 200 mg (400 mg the first time) and repeat it a maximum of twice a day as needed. Furthermore, they could take acetaminophen 200 mg, both when more than two hours had passed after taking the last medication and when they could not wait until the next celecoxib. They could repeat acetaminophen to a maximum of 4,000 mg (20 times) a day as needed. However, when more than six hours had passed from the last administration of celecoxib, they took celecoxib prior to acetaminophen. In addition, all patients took oral cefcapene pivoxil hydrochloride hydrate 300 mg a day postoperatively for three days.

Postoperative pain intensity, satisfaction with postoperative pain relief, adverse events, and postoperative supplemental analgesic rescue use (time, dose) were investigated using self-reports by the patients. Patients marked postoperative pain intensity on a line using a visual analog scale (VAS) at 4 h, 8 h, 24 h, 2 d, 3 d, 4 d, 5 d, 6 d, and 7 d after tooth extraction, and satisfaction was evaluated using a five-grade score (1 “dissatisfied”, 2 “rather dissatisfied”, 3 “neither”, 4 “rather satisfied”, and 5 “satisfied”) at 24 h, 2 d, 3 d, 4 d, 5 d, 6 d, and 7 d after tooth extraction. Patients also recorded the time and dose of analgesic use and all adverse events through 1 week.

In addition, lidocaine plasma concentration levels were measured at 24 h and 7 d after tooth extraction. All subjects underwent a blood examination at 14 d after tooth extraction to detect serious side effects such as liver injury, renal injury, or pancytopenia. Medical interviews or examinations were also performed in the hospital at 24 h, 7 d (to remove sutures), and 14 d, or as needed (Table 1).

**Table 1:** Schedule.

	Pre-	Enroll	4 h	8 h	24 h	2 d to 6 d	7 d	14 d
Informed consent	○							
Tooth extraction		○						
VAS			○	○	○	○	○	
Analgesic rescue			○	○	○	○	○	
Satisfaction					○	○	○	
Hospital visit	○	○			○	As needed	○	○
Lidocaine concentration	○				○		○	
Blood examinations	○							○
ECG	○							

### Preparation of the study drug

**Fig 5:**
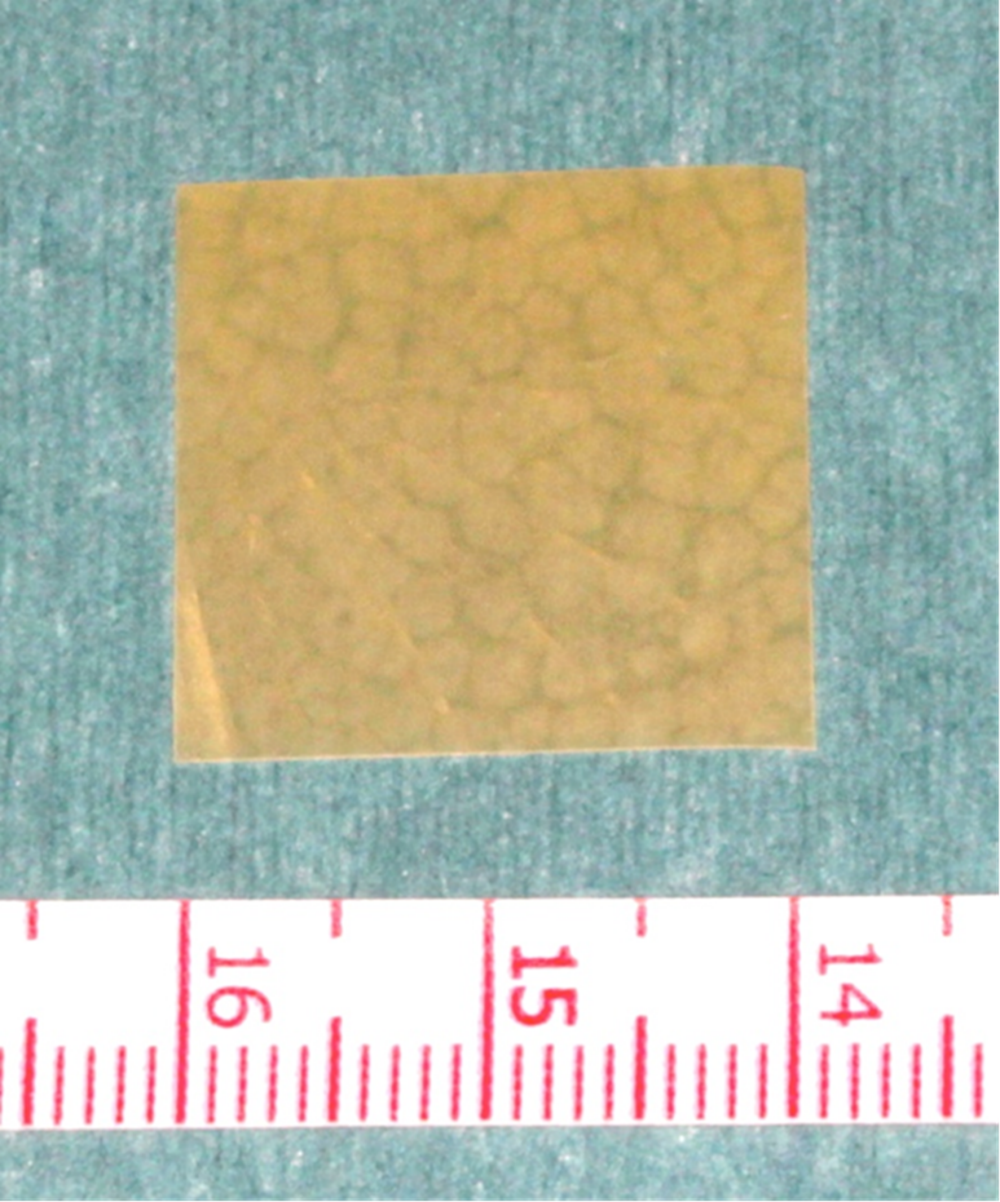
Sustained-release lidocaine sheet. The sustained-release lidocaine sheet 100 mg is approximately 2-cm square, with a thickness of approximately 0.5 mm and high plasticity. It has a white particle of lidocaine inside.

SRLSs loaded with 40% (w/w) lidocaine were prepared twice before this clinical study (Fig 5) and mixed together. Lidocaine (804.1 mg; lidocaine powder; Sigma-Aldrich Corporation, St. Louis, MO) and PLGA (1,204 mg; 50:50 poly (DL-lactide-co-glycolide) ester-terminated polymer; inherent viscosity, 0.55–0.75; Durect Corporation, Cupertino, CA, USA) were dissolved in chloroform (6.310 ml; ≥99.5%; containing 100–200 ppm amylenes as a stabilizer; 1.492 g/mL at 25°C; Sigma-Aldrich Corporation), and then the lidocaine/PLGA/chloroform solution was poured into Petri dishes (inner diameter 48 mm, area approximately 7616 mm^2^). The solutions were then desiccated for 2 days at 25°C in a class II, type A2 biological safety cabinet (Thermo Fisher Scientific Inc., Waltham, MA, USA) whose interior had been sterilized with a germicidal light, followed by 1 week at 37°C in a vacuum-drying oven (Advantec Toyo Kaisha, Ltd., Bunkyo, Tokyo, Japan) to allow the chloroform to evaporate completely. The drying converted the solutions to sheets, which were removed from the dishes and cut into pieces of approximately 2 cm × 2 cm, each weighing approximately 100 mg and containing approximately 40 mg of lidocaine. The prepared SRLS samples were sterilized in vacuo by 25-kGy gamma radiation (60 Co) by Radie Industry Co., Ltd. (Takasaki, Gunma, Japan) and frozen to be stored until use.

The capacity of the SRLS to release lidocaine in vitro was determined before the study. SRLS samples were placed into a vessel filled with 50 mL of phosphate buffer (pH 7.40) prepared from monobasic sodium phosphate (12.69 g) and dibasic sodium phosphate (43.74 g) in 4 L of distilled water. Four vessels were prepared in the same way, and 0.5 ml of the buffer was sampled from each of the vessels at several time points. The vessels were placed in a 37°C incubator except when sampled. The lidocaine concentration in each sample was measured by SRL Inc. (Shinjuku, Tokyo, Japan) using an enzyme immunoassay (EIA).

### Statistics

All statistical analyses were performed with EZR (Saitama Medical Center, Jichi Medical University, Saitama, Japan), which is a graphical user interface for R (The R Foundation for Statistical Computing, Vienna, Austria, Version 3.3.2). More precisely, it is a modified version of R commander (version 2.3–0) designed to add statistical functions frequently used in biostatistics [16].

The analysis was not adjusted for differences at baseline. The significance of differences among parametric data comparisons between groups was evaluated using one-way analysis of variance (ANOVA) and repeated measures ANOVA. The significance of differences among ordinal data comparisons between groups was evaluated using the Kruskal-Wallis test. The significance of differences among proportional comparisons between groups was evaluated using Fisher’s exact test. When significant differences were observed, P values were adjusted using the Benjamini and Hochberg method [17] for between-group comparisons. A value of P<0.05 was considered significant.

## Results

The patients in this trial were enrolled between January 2014 and December 2016. Patient disposition is shown in Fig 1. A total of 113 patients were enrolled, 99 were randomized, and 14 were excluded. Five patients did not meet the inclusion criteria, 2 declined to participate, 2 could not find a suitable time, 1 had abnormally high blood pressure on the day of the extraction, and 4 were excluded because of other reasons. Of the 99 patients randomized, 94 (94.9%) completed the study and were analyzed as the efficacy assessment population. Two patients did not visit a hospital, and questionnaire sheets could not be collected from 2 patients. One was also excluded because of postoperative bleeding. The patient with postoperative bleeding and the 94 patients who completed the study, 95 patients in all, were analyzed as the safety assessment population. Demographics and baseline characteristics were similar across the 5 groups in the efficacy assessment population (Table 2) and the safety assessment population.

**Table 2:** Demographics and baseline characteristics (the efficacy assessment population).

			Groups					
			None	PLGA 100 mg	SRLS 100 mg	SRLS 200 mg	SRLS 400 mg	

Efficacy assessment population			N = 20	N = 17	N = 20	N = 18	N = 19	P values
Height	(cm)	mean±SD	167±8	165±8	164±9	167±9	164±6	P = 0.505^†^
Weight	(kg)	mean±SD	62±14	63±13	58±9	62±12	60±10	P = 0.721^†^
BMI	(kg/m^2^)	mean±SD	22±3	23±4	22±2	22±3	22±3	P = 0.868^†^
Age	(y)	mean±SD	27±7	31±7	28±9	31±8	33±8	P = 0.0945^†^
Local Anesthetic Dose	(ml)	mean±SD	3.6±0.0	4.1±1.0	3.8±0.6	3.9±0.6	3.9±0.7	P = 0.231^†^
Operative time	(min)	mean±SD	9±4	11±7	11±8	10±4	11±5	P = 0.698^†^
Sutures		mean±SD	2±0	2±1	2±0	2±0	3±2	P = 0.155^†^
Sex	Male	N (%)	12(60)	8(47)	8(40)	10(56)	8(42)	P = 0.691^‡^
	Female	N (%)	8(40)	9(53)	12(60)	8(44)	11(58)	
ASA-PS	1	N (%)	11(55)	14(82)	17(89)	12(67)	13(72)	P = 0.141^‡^
	2	N (%)	9(45)	3(18)	2(11)	6(33)	5(28)	
Place	Left	N (%)	11(55)	7(41)	12(60)	6(33)	10(53)	P = 0.486^‡^
	Right	N (%)	9(45)	10(59)	8(40)	12(67)	9(47)	
Pell & Gregory's Class	1	N (%)	4(20)	2(12)	3(15)	4(22)	1(5)	P = 0.501^‡^
	2	N (%)	16(80)	12(71)	16(80)	13(72)	15(79)	
	3	N (%)	0	3(18)	1(5)	1(6)	3(16)	
Pell & Gregory's Position	A	N (%)	8(40)	7(41)	6(30)	5(28)	4(21)	P = 0.569^‡^
	B	N (%)	12(60)	9(53)	14(70)	13(72)	15(79)	
	C	N (%)	0	1(6)	0	0	0	

^†^One-way ANOVA

^‡^Fisher’s exact test

### Postoperative pain intensity

**Fig 6:**
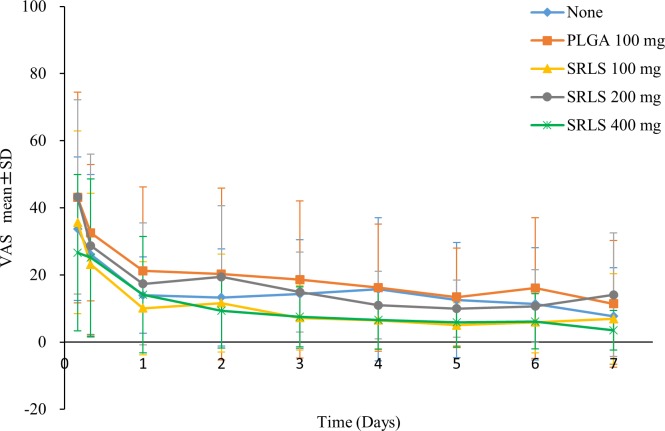
VAS scores for postoperative pain. The efficacy or the dose-response of SRLS was evaluated by comparing the VAS scores across the non-administration, PLGA Control, SRLS 100 mg, 200 mg, and 400 mg groups.

The visual analogue scale (VAS) scores were calculated by measuring with a ruler. The efficacy or the dose-response of the sustained-release lidocaine sheet (SRLS) was evaluated by comparing the VAS scores at each time point after tooth extraction (Fig 6 and S1 Table). There was no significant difference (P = 0.1348, repeated measures analysis of variance: ANOVA) in the VAS scores among the 5 groups.

### Satisfaction with postoperative pain relief

**Fig 7:**
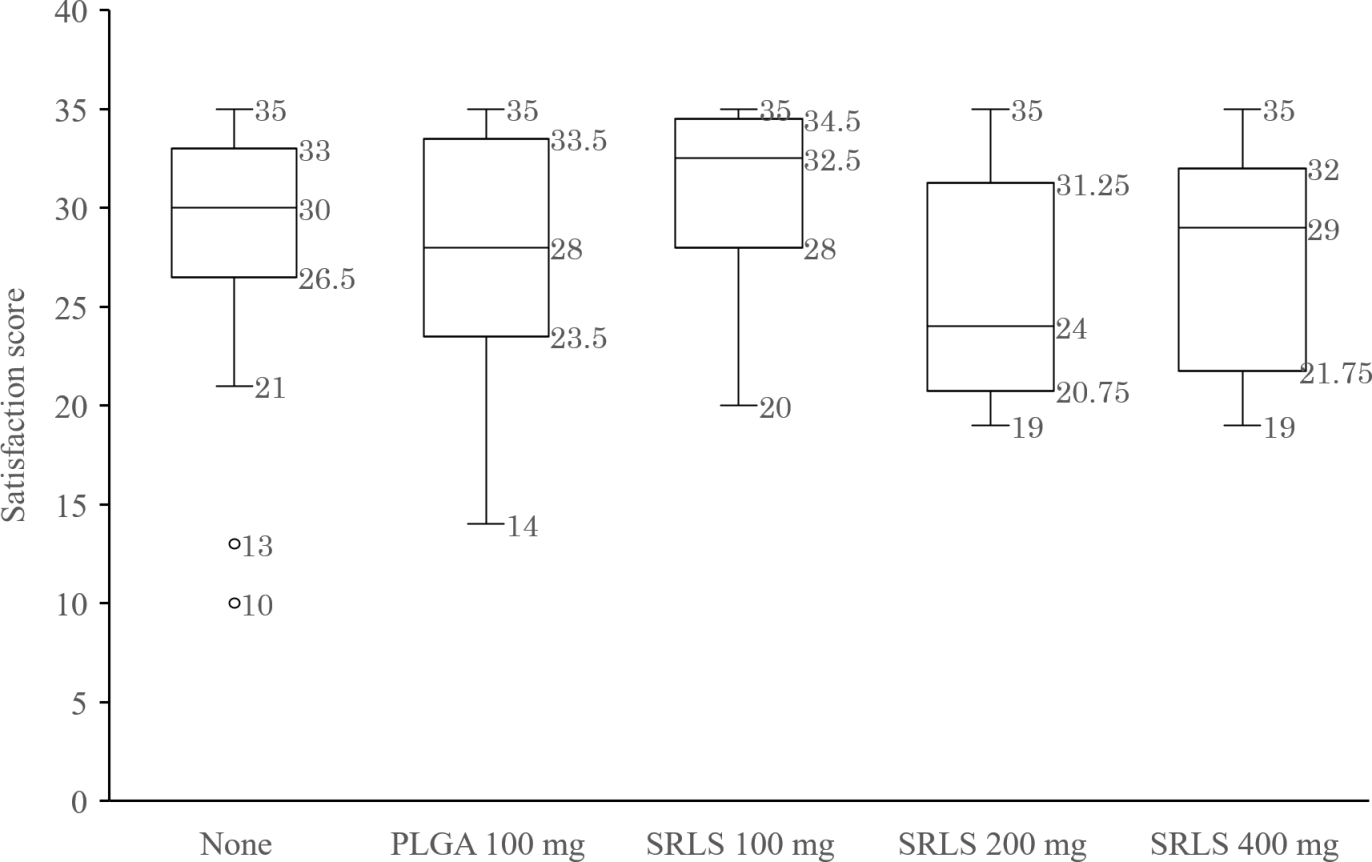
Satisfaction with postoperative pain relief. The efficacy or the dose-response of the SRLS was assessed by comparing the Satisfaction scores across the non-administration, PLGA Control, and SRLS 100 mg, 200 mg, and 400 mg groups.

The total satisfaction score for a week was calculated by adding the 7 values at 24 h, 2 d, 3 d, 4 d, 5 d, 6 d, and 7 d. Patients with missing values for satisfaction were excluded from the calculation. The efficacy or the dose-response of the SRLS was evaluated by comparing the Satisfaction scores (Fig 7). The median (maximum/minimum) satisfaction scores in the non-administration, PLGA Control, SRLS 100 mg, 200 mg, and 400 mg groups were 30 (35/21), 28 (35/14), 32.5 (35/20), 24 (35/19), and 29 (35/19), respectively. There was no significant difference in the satisfaction scores among the 5 groups (P = 0.139, Kruskal-Wallis test).

### Postoperative supplemental analgesic rescue use

**Fig 8:**
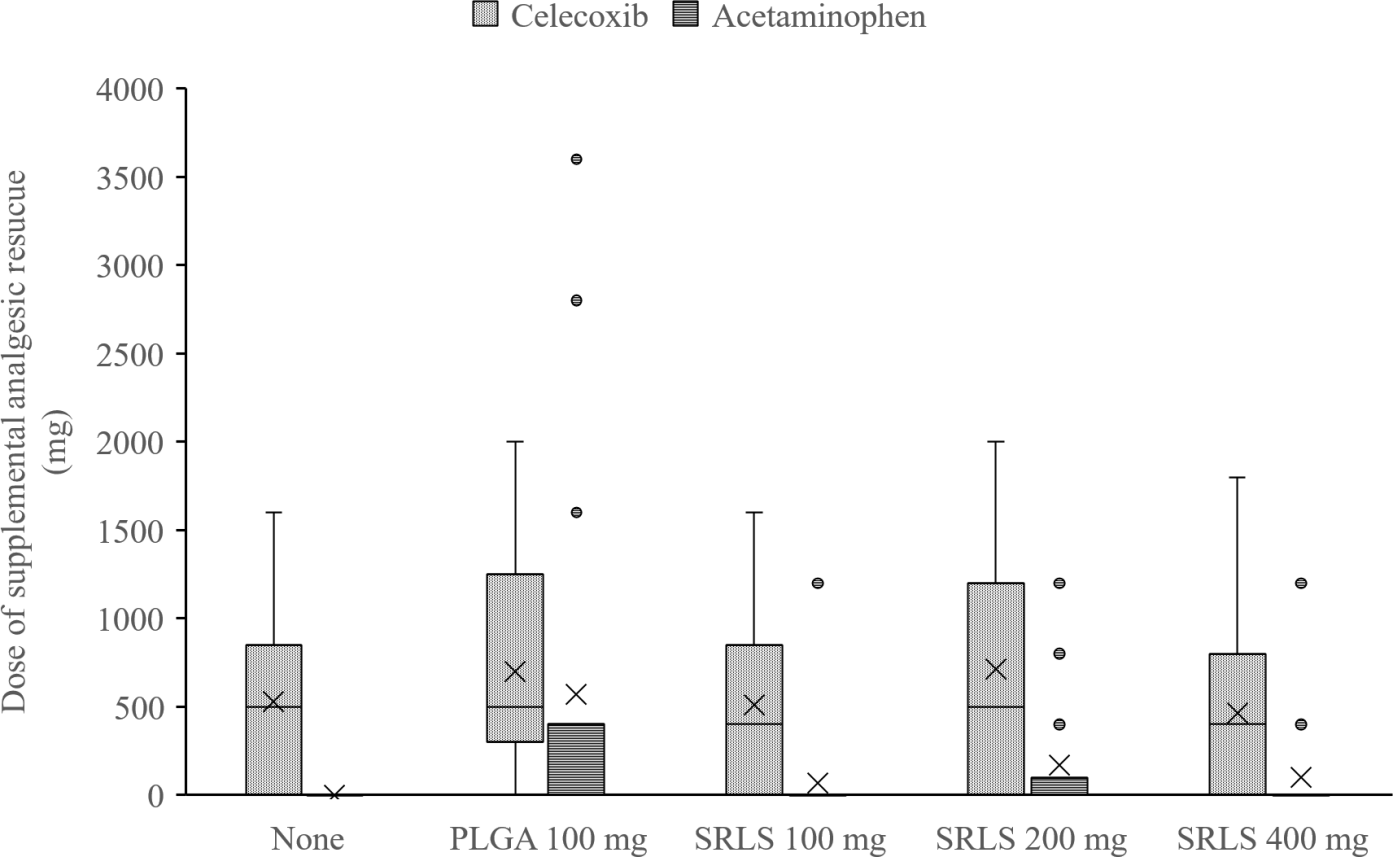
Total dose of supplemental analgesic rescue. The efficacy or the dose-response of SRLS was evaluated by comparing the dose of supplemental analgesic rescue across the non-administration, PLGA Control, SRLS 100 mg, 200 mg, and 400 mg groups.

**Fig 9:**
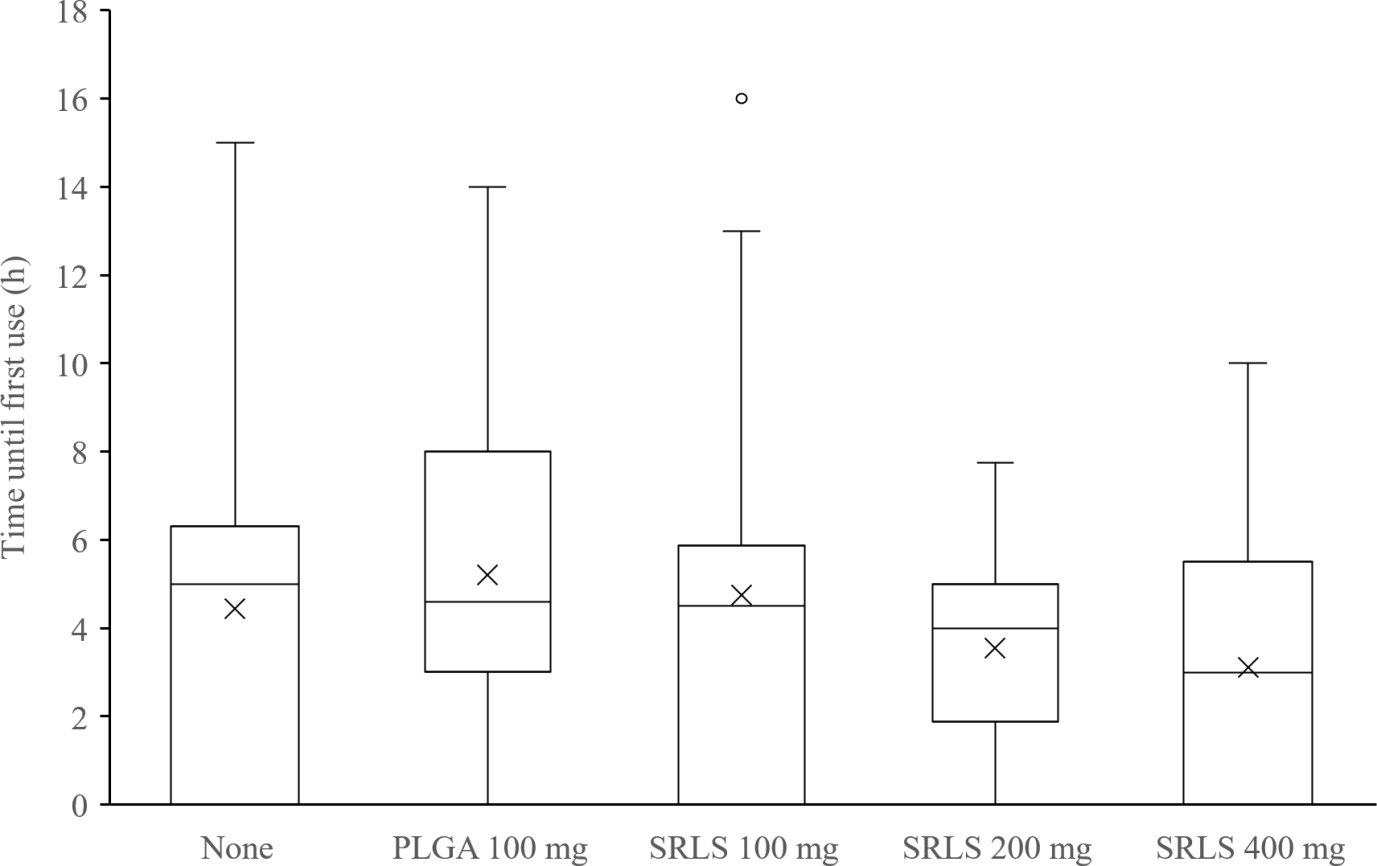
Time until first use of supplemental analgesic rescue. The efficacy or the dose-response of SRLS was evaluated by comparing the time until first use across the non-administration, PLGA Control, SRLS 100 mg, 200 mg, and 400 mg groups.

**Fig 10:**
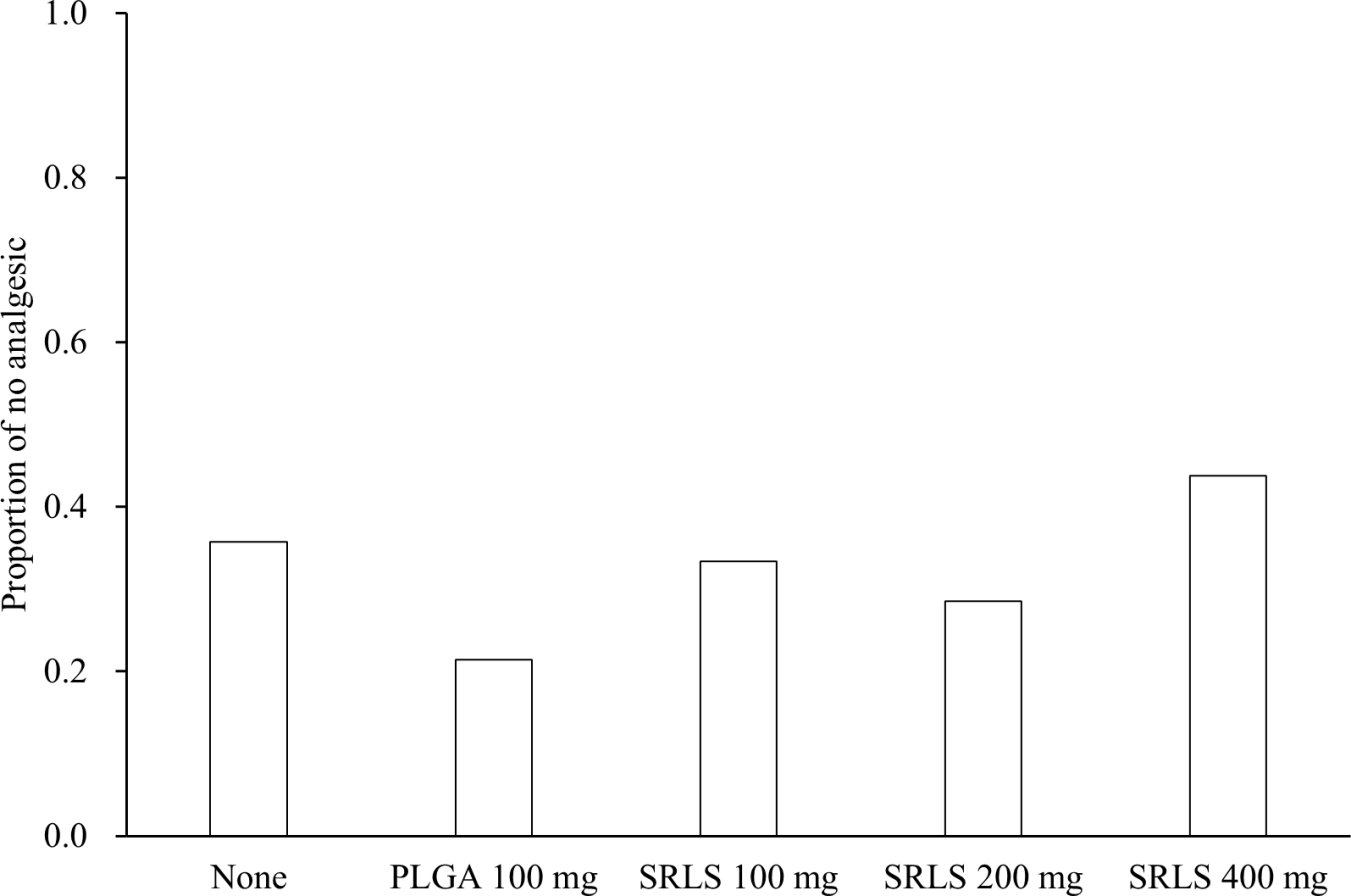
Proportion of patients who took no analgesic rescue. The efficacy or the dose-response of the SRLS was evaluated by comparing the proportion of no analgesic across the non-administration, PLGA Control, SRLS 100 mg, 200 mg, and 400 mg groups.

The total dose of supplemental analgesic rescue, the time until first use of supplemental analgesic rescue, and the proportion of patients who took no analgesic rescue were calculated. The efficacy or the dose-response of the SRLS was evaluated by comparing the dose of supplemental analgesic rescue (Fig 8), the time until first use (Fig 9), and the proportion of no analgesic use (Fig 10). The mean (SD) doses of celecoxib in the non-administration group, the PLGA 100 mg control group, the SRLS 100 mg group, the SRLS 200 mg group, and the SRLS 400 mg group were 529 (512) mg, 700 (611) mg, 511 (500) mg, 714 (699) mg, and 463 (525) mg, respectively. The mean (SD) doses of acetaminophen in the non-administration group, PLGA 100 mg control group, SRLS 100 mg group, SRLS 200 mg group, and SRLS 400 mg group were 0 (0) mg, 571 (1202) mg, 67 (283) mg, 171 (375) mg, and 100 (310) mg, respectively. The mean (SD) times until first use in the non-administration group, PLGA 100 mg control group, SRLS 100 mg group, SRLS 200 mg group, and SRLS 400 mg group were 5.6 (3.3) h, 6.3 (3.1) h, 6.8 (3.8) h, 4.1 (1.5) h, and 4.9 (2.4) h, respectively. The proportions of no analgesic use in the non-administration group, PLGA 100 mg control group, SRLS 100 mg group, SRLS 200 mg group, and SRLS 400 mg group were 35.7%, 21.4%, 33.3%, 28.6%, and 43.8%, respectively. There were no significant differences in the dose of celecoxib (P = 0.644, one-way ANOVA), the dose of acetaminophen (P = 0.0753, one-way ANOVA), the time until first use (P = 0.273, one-way ANOVA), and the proportion of no analgesic use (P = 0.788, Fisher’s exact test) among the 5 groups.

### Adverse events

The incidence of AEs stratified by group is summarized in Table 3. Overall, 64 (67.4%) of the 95 patients who received study drug reported at least one adverse event (AE) during the study: 12 (60.0%) in the non–administration group, 13 (72.2%) in the PLGA Control group, 10 (50.0%) in the SRLS 100 mg group, 13 (72.2%) in the SRLS 200 mg group, and 16 (84.2%) in the SRLS 400 mg group (P = 0.203, Fisher’s exact test). There were no significant differences in each AE except for trismus (P = 0.0148, Fisher’s exact test). However, there were no significant differences in the adjusted P values of trismus among the 5 groups (S2 Table).

**Table 3:** Adverse events.

	Groups					
	None	PLGA 100 mg	SRLS 100 mg	SRLS 200 mg	SRLS 400 mg	
Safety assessment population	N = 20	N = 18	N = 20	N = 18	N = 19	Fisher’s exact test
	N(%)	N(%)	N(%)	N(%)	N(%)	
≥1 AE	12(60.0)	13(72.2)	10(50.0)	13(72.2)	16(84.2)	P = 0.203
Swelling	5(25.0)	5(27.8)	4(20.0)	4(22.2)	8(42.1)	P = 0.609
Trismus*	5(25.0)	5(27.8)	0	1(5.6)	6(31.6)	P = 0.0148
Bitterness	0	1(5.6)	2(10.0)	3(16.7)	5(26.3)	P = 0.079
Odontorrhagia	1(5.0)	2(11.1)	1(5.0)	2(11.1)	0	P = 0.540
Subcutaneous hemorrhage	3(15.0)	1(5.6)	0	3(16.7)	3(15.8)	P = 0.315
Dry socket	1(5.0)	3(16.7)	3(15.0)	1(5.6)	0	P = 0.304
Numbness	1(5.0)	1(5.6)	1(5.0)	1(5.6)	2(10.5)	P = 0.946
Large granulation	0	0	0	1(5.6)	1(5.3)	P = 0.418
Mouth ulcer	2(10.0)	0	0	0	1(5.3)	P = 0.450
Sore throat	0	0	0	1(5.6)	1(5.3)	P = 0.418
Sore tongue	0	1(5.6)	1(5.0)	0	1(5.3)	P = 0.841
Difficulty chewing	2(10.0)	1(5.6)	1(5.0)	0	1(5.3)	P = 0.958
Tongue-tied	0	1(5.6)	0	0	0	P = 0.379
Fever	2(10.0)	0	1(5.0)	2(11.1)	0	P = 0.457
Headache	0	0	1(5.0)	1(5.6)	1(5.3)	P = 0.841
Anxiety	0	0	0	1(5.6)	0	P = 0.379
Sleepiness	0	0	0	0	1(5.3)	P = 0.579
Nausea	0	0	0	1(5.6)	0	P = 0.379
Bowel distention	0	1(5.6)	0	0	0	P = 0.379
Creatinine elevated	0	1(5.6)	1(5.0)	0	0	P = 0.740
T-Bil elevated	0	0	0	0	1(5.3)	P = 0.579
CK elevated	0	0	1(5.0)	0	0	P = 1.000
K elevated	1(5.0)	0	0	0	0	P = 1.000
ALT elevated	0	1(5.6)	0	0	0	P = 0.379
WBC elevated	1(5.0)	0	0	0	1(5.3)	P = 0.91

Fisher’s exact test *P<0.05

The most frequently reported AEs were swelling, trismus, and bitter taste. Five patients had serious AEs during the study. In the PLGA 100 mg control group, one patient had odontorrhagia on the day of the surgery, and one patient had postoperative ALT elevation (from 27 to 34 U/l). The first received hemostasis and elimination of the remaining study drug as an emergency and discontinued participation in the study. Furthermore, the first patient also had postoperative creatinine elevation (from 0.69 to 0.80 mg/dl). The latter was followed-up, and ALT was re-examined one month later, and recovery was confirmed. Another patient in the SRLS 100 mg group also had postoperative creatinine elevation (from 0.77 to 0.82 mg/dl) and needed to be re-examined, but it was not possible to make contact with her. Two patients had postoperative WBC elevations: one (WBC 7000 to 9800/μl) in the SRLS 400 mg group was followed-up and re-examined 3 weeks later, and then recovery was confirmed; one (WBC 5,300 to 10,500/μl) in the non–administration group was also followed-up.

No subjects reported symptoms of local anesthetic toxicity. In addition, lidocaine plasma concentration levels were lower than the detectable threshold at 24 h and 7 d after tooth extraction in all patients who received the SRLS.

### In vitro release of lidocaine from the SRLS

**Fig 11:**
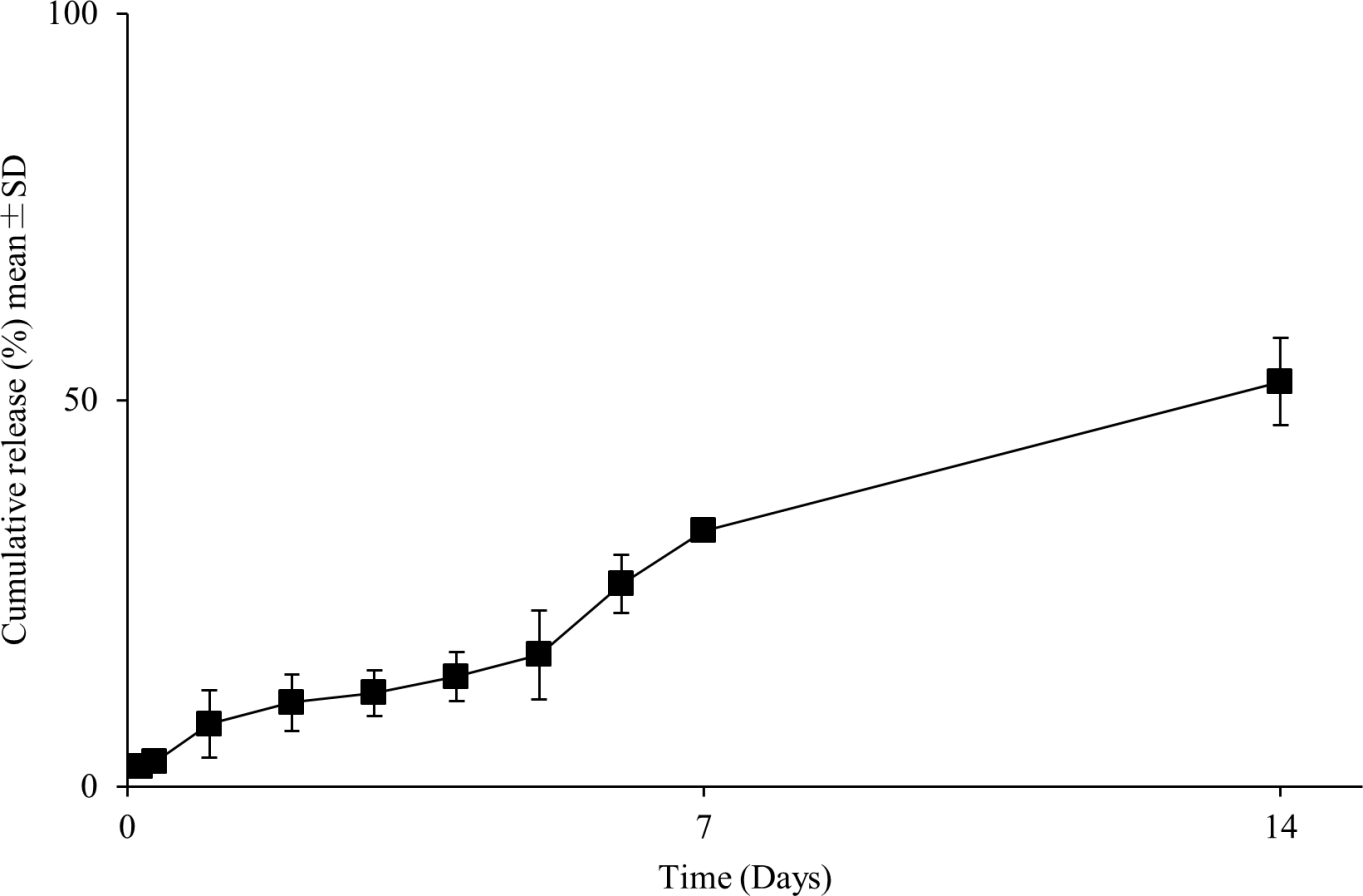
In vitro release of lidocaine from the SRLS. The SRLS shows approximately linear release of lidocaine for more than the 7 d administration period in this clinical study. The fraction of lidocaine released from the SRLS relative to the actual lidocaine content in the SRLS is shown as the mean±standard deviation (n = 4).

The cumulative release of lidocaine from the SRLS into phosphate buffer was calculated (Fig 11). The SRLS showed approximately linear release of lidocaine over 2 w. The mean (SD) release rates of lidocaine from the SRLS at 4 h, 8 h, 24 h, 2 d, 3 d, 4 d, 5 d, 6 d, 7 d, and 14 d were 2.6 (0.8)%, 3.2 (0.7)%, 8.1 (4.3)%, 10.9 (3.7)%, 12.1 (2.9)%, 14.2 (3.2)%, 17.0 (5.7)%, 26.2 (3.8)%, 33.1 (0.9)%, and 52.4 (5.6)%, respectively.

## Discussion

This was the first clinical trial of the SRLS in patients. This randomized, single-blind, dose-response, controlled, clinical study was meant as an initial intervention in ultimately developing and refining the sustained-release lidocaine particles (SRLPs) for administration into the epidural space or around sensory nerves, and it aimed to evaluate the efficacy, safety, and appropriate dose of the SRLS for pain following tooth extraction (Phase 1/2 clinical study). However, there were no significant differences in the efficacy and dose-response study across the groups. It is possible that the pain following tooth extraction was originally not very severe, or the number of cases was too small to compare across the groups. Sample size was analyzed in each group of the present study using one-way analysis of variance (ANOVA) with 5 groups, 80% power to detect, and a significance level of 0.05. The sample sizes for the VAS scores at 4 h, 8 h, 24 h, 2 d, 3 d, 4 d, 5 d, 6 d, and 7 d after tooth extraction were 55, 156, 68, 53, 34, 36, 36, 43, and 52, respectively. Therefore, the sample size would be larger when repeated measures ANOVA is used more accurately with 5 groups.

No subjects reported symptoms of local anesthetic toxicity, and lidocaine plasma concentration levels were lower than the detectable threshold in all patients. However, the SRLS in this study released much less lidocaine than expected. According to Fig 11, the actually administered lidocaine was approximately 13.0 mg and 53.0 mg for 24 h and 7 d, respectively, in the maximum SRLS 400 mg group. The safety of the SRLS for patients undergoing tooth extraction was demonstrated, except for lidocaine toxicity. A further pharmacological study of the SRLS is needed.

Cumulative release of lidocaine from the SRLS in this study showed less release of lidocaine, with 14.2% released by 4 d, compared with 86.1% released by 4 d in the previous study [15], where the SRLS was not sterilized. In addition, less release of lidocaine might have had an effect on the lack of significant differences. The SRLS in the present study was sterilized by radiation, and then the PLGA matrix inside was broken down to become smaller molecules. The SRLS samples were melted and put together quickly to precipitate at the bottom of the vessel when cumulative release of lidocaine was calculated. Therefore, the surface area of the samples was considered to be decreased to release less lidocaine. Moreover, the properties of the SRLS are easily affected by the environment when it is made, and reproducibility was sometimes slightly poor. The properties of the SRLS may have been slightly different between batches because the SRLS was made twice for this study. Therefore, its variability might have had an effect on the lack of significant differences.

### Efficacy assessments

Efficacy showed no significant differences across the 5 groups, but the efficacy assessments were more closely examined across the non-administration, PLGA Control, and SRLS 100 mg groups (S1 Fig). The PLGA Control group showed the strongest pain, especially more than that of the non–administration group. This pain was probably caused by the filling pressure at the socket itself. In that regard, in the SRLS 100 mg group, the released lidocaine may have suppressed the pain derived from the filling pressure.

### Dose-response assessments

Dose-response showed no significant differences across the 5 groups, but the dose-response assessments were more closely examined across the SRLS 100 mg, 200 mg, and 400 mg groups (S2 Fig). The SRLS 200 mg group showed the strongest pain and the lowest total satisfaction score. As doses increased, the SRLS increased the filling pressure in the socket. Therefore, in the SRLS 200 mg group, the pain from the filling pressure probably overcame the analgesic action of the released lidocaine. On the other hand, in the SRLS 400 mg group, the analgesic action of the released lidocaine may be larger than the pain derived from the filling pressure. Consequently, the VAS score was equivalent to that in the 100 mg group and lower than that in the SRLS 200 mg group. Nevertheless, satisfaction in the SRLS 400 mg group was the same or slightly lower than that in the SRLS 100 mg group, probably due to discomfort from the filling pressure of the SRLS 100 mg×4 sheets or the bitterness caused by the released lidocaine. In the SRLS 100 mg group, analgesia by the released lidocaine and the negative influence from the filling pressure seemed to be balanced.

### Safety assessments

The incidence of adverse events (AEs) stratified by group is summarized in Table 3. There were no significant differences among the 5 groups at all, and five patients had serious AEs during the study. However, no clinically critical AEs considered related to study drug were observed. In the PLGA 100 mg control group, one patient had odontorrhagia and discontinued participation in the study. This odontorrhagia was considered to be caused by an unreported anticoagulant and unrelated to the study drug. Two patients had postoperative WBC elevations. These events were apparently caused by the surgical stress. The most frequently reported AEs were swelling, trismus, and bitterness. Swelling and trismus were caused by surgical inflammation, and bitterness was caused by the released lidocaine.

Accordingly, administration of the SRLS 100 mg may have clinical therapeutic potential for pain after tooth extraction. In addition, the analgesic effect may improve if more lidocaine is loaded into the same volume, so that filling pressure does not increase. The SRLS prepared for this study released much less lidocaine and could not provide any superior therapeutic effect when compared to both no treatment or a vehicle control. Furthermore, decreased visual analogue scale (VAS) scores by the SRLS should lead to decreasing consumption of analgesic, such as opioids, improving satisfaction as a result. The safety of the SRLS for patients undergoing tooth extraction was partially demonstrated, and the present study showed no significant differences in efficacy and dose-response across the groups. A larger sample size study of the SRLS with different causes of pain, including skin pain from heat burns or shingles, is needed.

## Conclusions

Sustained-release lidocaine using biodegradable polymers was applied as a sheet in patients undergoing tooth extraction for the first time. The sustained-release lidocaine sheet (SRLS) 100 mg may have clinical therapeutic potential for pain following tooth extraction, although there were no significant differences. There were no serious side effects, including plasma concentration increases of lidocaine, attributable to the SRLS in the present study.

## Supporting information

S1 Checklist CONSORT 2010 checklist of information to include when reporting a randomized trial. (DOC)

S1 Protocol Clinical trial protocol that the ethics committee approved (Original-Japanese). (DOC)

S2 Protocol Clinical trial protocol that the ethics committee approved (English-Translation). (DOC)

S1 Table VAS scores for postoperative pain in the non-administration, PLGA Control, SRLS 100 mg, 200 mg, and 400 mg groups. (DOCX)

S2 Table Adjusted P values for trismus. (XLSX)

S1 Fig VAS scores for postoperative pain in the non-administration, PLGA Control, and SRLS 100 mg groups. (TIF)

S2 Fig VAS scores for postoperative pain in the SRLS 100 mg, 200 mg, and 400 mg groups. (TIF)
