# Supplementary material for: Sustained-release lidocaine sheet for pain following tooth extraction: A randomized, single-blind, dose-response, controlled, clinical study of efficacy and safety
Source: PLoS One. 2018 Jul 2;13(7):e0200059. doi: 10.1371/journal.pone.0200059 (PMC6028143; doi:10.1371/journal.pone.0200059)
Supplement: S1 Protocol — (DOC) [file pone.0200059.s002.doc]

# **標題ページ**

**術後痛に対するリドカイン徐放シートの**

**Ⅰ/Ⅱ相臨床試験**

臨床研究分担医師

群馬大学医学部附属病院歯科口腔外科 　小杉謙介

臨床研究責任医師

群馬大学医学部附属病院麻酔科蘇生科　　鈴木敏之

臨床研究代表者

群馬大学医学部附属病院麻酔科蘇生科　　齋藤繁

作成年月日 ２０１３年７月０３日 版数： 第１版

２０１３年７月３０日 版数： 第２版

２０１３年７月３１日 第２．１版

２０１３年８月２０日 版数： 第３版

２０１３年８月２１日 版数： 第４版

２０１３年１０月２９日 第４．１版

２０１４年０１月２８日 第４．２版

２０１４年０３月２４日 第４．３版

２０１４年０８月１０日 第４．４版

**目次**

[**標題ページ** 1](#__RefHeading___Toc364775055)

[**略語および用語の定義** 4](#__RefHeading___Toc364775056)

[**（１）研究の背景及び研究実施の意義・必要性** 5](#__RefHeading___Toc364775057)

[**（２）研究の目的** 5](#__RefHeading___Toc364775058)

[**（３）本研究で用いる基準・定義** 5](#__RefHeading___Toc364775059)

[**（４）研究計画・研究デザイン** 5](#__RefHeading___Toc364775060)

[4-1　デザイン名 5](#__RefHeading___Toc364775061)

[4-2　研究の全般的なデザイン 6](#__RefHeading___Toc364775062)

[**（５）　患者（被験者）選択基準** 6](#__RefHeading___Toc364775063)

[5-1　適格基準 6](#__RefHeading___Toc364775064)

[5-2　除外基準 6](#__RefHeading___Toc364775065)

[**（６）治療等の介入の具体的内容** 6](#__RefHeading___Toc364775066)

[6-1　試験薬、医療機器、医用材料等の概要 6](#__RefHeading___Toc364775067)

[6-2　投与群 8](#__RefHeading___Toc364775068)

[6-3　治療等の介入を行う手順と経時的なスケジュール 8](#__RefHeading___Toc364775069)

[6-3-1　投与部位： 8](#__RefHeading___Toc364775070)

[6-3-2　治療等の介入を行う期間 8](#__RefHeading___Toc364775071)

[6-3-3　用法・用量 8](#__RefHeading___Toc364775072)

[6-3-4　増量・減量の目安等 8](#__RefHeading___Toc364775073)

[6-3-5　併用薬及び併用療法 9](#__RefHeading___Toc364775074)

[**（７）観察・検査・調査項目及び実施時期** 9](#__RefHeading___Toc364775075)

[7-1　観察・検査項目 9](#__RefHeading___Toc364775076)

[7-2　観察・検査方法 10](#__RefHeading___Toc364775077)

[7-3　実施時期・スケジュール 10](#__RefHeading___Toc364775078)

[**（８）重篤な有害事象への対応** 10](#__RefHeading___Toc364775079)

[**（９）研究の中止基準** 10](#__RefHeading___Toc364775080)

[9-1　患者（被験者）ごとの中止基準 10](#__RefHeading___Toc364775081)

[9-2　研究全体の中止基準 10](#__RefHeading___Toc364775082)

[**（１０）患者（被験者）の登録方法・割付方法** 10](#__RefHeading___Toc364775083)

[**（１１）研究実施期間** 11](#__RefHeading___Toc364775084)

[**（１２）予定症例数** 11](#__RefHeading___Toc364775085)

[**（１３）統計学的事項** 11](#__RefHeading___Toc364775086)

[13-1　有効性評価項目 11](#__RefHeading___Toc364775087)

[13-1-1　主要評価項目（プライマリーエンドポイント） 11](#__RefHeading___Toc364775088)

[13-1-2　副次的評価項目（セカンダリーエンドポイント） 11](#__RefHeading___Toc364775089)

[13-2　安全性評価項目 11](#__RefHeading___Toc364775090)

[13-3　解析方法 11](#__RefHeading___Toc364775091)

[13-4　中間解析と研究の早期中止 11](#__RefHeading___Toc364775092)

[**（１４）臨床試験審査委員会への報告義務** 11](#__RefHeading___Toc364775093)

[**（１５）症例報告書の取り扱い** 12](#__RefHeading___Toc364775094)

[**（１６）倫理的事項** 12](#__RefHeading___Toc364775095)

[16-1　遵守すべき諸規則 12](#__RefHeading___Toc364775096)

[16-2　インフォームド・コンセントの手順 12](#__RefHeading___Toc364775097)

[16-3　同意説明文書の内容 12](#__RefHeading___Toc364775098)

[16-4 研究内容の公開 12](#__RefHeading___Toc364775099)

[16-5 患者（被験者）の個人情報及びプライバシーの保護 12](#__RefHeading___Toc364775100)

[**（１７）健康被害に対する補償・賠償** 13](#__RefHeading___Toc364775101)

[**（１８）予測される医療費（患者（被験者）の負担）** 13](#__RefHeading___Toc364775102)

[**（１９）患者（被験者）に対する金銭の支払、医療費の補助** 13](#__RefHeading___Toc364775103)

[**（２０）研究資金の拠出元** 13](#__RefHeading___Toc364775104)

[**（２１）利益相反** 13](#__RefHeading___Toc364775105)

[**（２２）研究実施計画書の改訂** 13](#__RefHeading___Toc364775106)

[**（２３）研究に関する資料等の利用と保存** 13](#__RefHeading___Toc364775107)

[**（２４）特記事項** 13](#__RefHeading___Toc364775108)

[**（２５）研究成果の帰属と結果の公表** 13](#__RefHeading___Toc364775109)

[**（２６）研究組織及び連絡先** 14](#__RefHeading___Toc364775110)

[**（２７）参考資料、文献リスト、付録** 14](#__RefHeading___Toc364775111)

**略語および用語の定義**

本研究実施計画書に使用する略語及び用語を下記に示す。

------------------------------------------------------------------------------

略語・専門用語 用語の説明

------------------------------------------------------------------------------

ANOVA 分散分析; Analysis Of Variance

ALT アラニンアミノトランスフェラーゼ; Alanine Aminotransferase

AST アスパラギン酸アミノトランスフェラーゼ; Aspartate Aminotransferase

ASA 米国麻酔学会; American Society of Anesthesiologists

AUC 曲線下面積; Area Under the Curve

GCP 医薬品の臨床試験の実施の基準; Good Clinical Practice

IV インヘレント粘度（対数粘度数）; Inherent Viscosity

NSAIDs 非ステロイド性抗炎症薬; Non-Steroidal Anti-Inflammatory Drugs

PLGA ポリ(ラクチド-co-グリコリド)共重合体; Poly (Lactic-co-Glycolic Acid)

PS 術前状態Physical Status

SRLS リドカイン徐放シート; Slow- Release Lidocaine Sheet

VAS 視覚的評価スケール; Visual Analog Scale

------------------------------------------------------------------------------

**（１）研究の背景及び研究実施の意義・必要性**

近年術後鎮痛への関心が高まり、様々な鎮痛薬により様々な鎮痛法が手術内容や患者リスク等に応じて用いられている。しかし、現在においても術後鎮痛に関しては充分満足のいく状況とは言えない。たとえば、抗凝固薬や抗血小板薬内服患者の手術が増加したり、また術後早期から抗凝固療法を開始したりすることが増え、硬膜外麻酔法は血腫による神経障害発生のリスクから減少傾向にある。代わって増えているのがオピオイドを静脈内持続投与する方法だが、オピオイドは体動時痛に対する効果が弱く、さらに嘔気や嘔吐、眠気、呼吸抑制のような副作用の発現が、十分な鎮痛の妨げになることがある。最近では機器の解像度の改善とともに超音波ガイド下末梢神経ブロック法が増えていて、安全で非常に良い鎮痛方法ではあるが、技術取得の必要性や初期コストの大きさ等の問題がある。そして、長時間作用を狙うにはどれをとっても持続投与が必要となる。そこで局所麻酔薬を長時間にわたり少しずつ放出（徐放）させることができれば、手術創部や支配神経周囲への単回投与によって、体動時でも効果的でかつ副作用の少ない、安全な術後鎮痛を施すことができると考えた。

　当研究室では2010年に生体内分解材料（体内で分解・吸収され最終的に水と二酸化炭素に分解される。実際、吸収性縫合糸等としても既に臨床で使われているもの）を用いてリドカイン徐放シートを作成し、ラットの坐骨神経ブロックでその長時間作用と安全性を確認した^1）^。その後、当施設での倫理委員会の許可を得てリドカイン徐放シートはヒトボランティアに対し、正常粘膜の外用での使用において安全性と長時間作用を確認した^2）^。世界的にみると、局所麻酔徐放薬として、臨床応用に至っているものはブピバカインのリポソームのみである^3）^。しかしリドカイン徐放シートとは剤形を異にするものであり、この剤形で臨床応用されているものは世界的に全く報告されていない。よって数日から数週間鎮痛効果を発揮するこれらの薬剤が開発されれば、非常に独創性の高い研究となる。痛みの治療は社会的意義が大きく、効果的でかつ副作用の少ない局所麻酔徐放薬が開発されれば、医学の進歩のみならず医療経済への貢献は大きいと思われる。

**（２）研究の目的**

　抜歯後患者においてリドカイン徐放シートを適応し、抜歯後の痛みに対する安全性（中毒量と投与量の検討）と有効性の確認を行う。

**（３）本研究で用いる基準・定義**

　視覚的評価スケール（VAS）：　「0」を「痛みはない」状態、「100」を「これ以上の痛みはないくらい痛い（これまで経験した一番強い痛み）」状態として、現在の痛みが10cmの直線上のどの位置にあるかを示す方法で痛みを評価する。診療の場で最も多く使われる。

　患者満足度：　「１」を「不満」、「２」を「どちらかというと不満」、「３」を「普通」、「４」を「どちらかというと満足」、「５」を「満足」状態として、満足度を5段階で評価する。

　米国麻酔学会術前状態（ASA PS）分類：　手術患者の全身状態を6クラスに分類し、この手術前における評価と患者の予後は相関するとされる。

ASA PS 1 - A normal healthy patient

ASA PS 2 - A patient with mild systemic disease

ASA PS 3 - A patient with severe systemic disease

ASA PS 4 - A patient with severe systemic disease that is a constant threat to life

ASA PS 5 - A moribund patient who is not expected to survive without the operation

ASA PS 6 - A declared brain-dead patient whose organs are being removed for donor purposes

# **（４）研究計画・研究デザイン**

## 4-1　デザイン名

Ⅰ/Ⅱ相臨床試験（単盲検無作為割り付け試験）

## 4-2　研究の全般的なデザイン

同意取得

登録

終了時検査

投与群Ｃ～Ｅ

Ａ非投与群

無作為化割付

Ｂ対照群

登録前検査

| **群馬大学医学部附属病院麻酔科蘇生科** | **Padjadjaran University** |
| --- | --- |

| 試験薬の作成 |
| --- |

| 臨床試験の実施・データ採取 | 臨床試験の実施・データ採取 |
| --- | --- |

（当該IRBにて承認取得予定）

| データ解析・処理 |
| --- |

| 共同研究として公に発表 |
| --- |

# **（５）　患者（被験者）選択基準**

下記の適格基準を全て満たし、かつ除外基準のいずれにも該当しない患者（被験者）を対象とする。

## 5-1　適格基準

１）20歳以上50歳未満の患者

２）男女外来患者

３）予定手術患者

４）米国麻酔学会術前状態分類のPS 1または2に相当する患者

５）下顎水平埋伏智歯の抜歯術を受ける患者

６）本研究の参加に関して同意が文書で得られている患者

## 5-2　除外基準

１）鎮痛に関与すると思われる薬物（抗精神病薬、NSAIDs、オピオイドなど）を服用中の患者、または中止されていてもその影響が残っていると判断される患者

２）手術部位が著しく感染している患者

３）リドカイン又はアミド型局所麻酔薬、セレコキシブ又はスルホンアミド、アセトアミノフェンに対し過敏症の既往歴のある患者

４）重篤な刺激伝導障害（完全房室ブロック等）のある患者

５）喘息、消化性潰瘍のある患者

６）意識障害もしくは意思疎通が困難で正当な評価を得られない患者

７）その他、医師の判断により対象として不適当と判断された患者

# **（６）治療等の介入の具体的内容**

6-1　試験薬、医療機器、医用材料等の概要

試験薬：　40%リドカイン徐放シート

材料：　PLGA (50:50), IV 0.55-0.75;

DURECT, LACTEL® Absorbable Polymers, 50:50 Poly (DL-lactide-co-glycolide) Ester Terminated Polymers, Inherent Viscosity 0.55-0.75, Product No: B6010-2^4）^

リドカイン（塩酸塩で無いもの）;

SIGMA-ALDRICH, Lidocaine powder, Product No: L7757^5）^

作成方法：

1. 材料を小ビンに入れクロロホルム^6）^で溶解する。
2. 溶解確認後、水平台にのせたPETRI DISH（シャーレ）に撒く。
3. 使用前最低２時間殺菌灯照射後の安全キャビネット内で蓋をせずに２日間常温（26℃程度）で溶媒を蒸発させる。
4. 蓋をして真空乾燥器内でさらに１４日間、３７～４０℃程度で溶媒を蒸発させる。
5. γ線滅菌
6. 冷凍保存（－20℃以下）

成分：　試験薬100㎎あたりリドカイン40㎎（クロロホルム10㎎以下）

徐放性能：　in vitroにおける徐放曲線 Mean±SD （n= 4）。40%リドカイン徐放シート（50:50, PLGA, IV 0.55-0.75)。

血行動態^1）^：　ラット（Sprague-Dawley）（250-300g）における血清リドカイン濃度 Mean±SD （n= 6 in each group）。30％リドカイン徐放シート20㎎（リドカイン6㎎）（50:50, PLGA, molecular weight 85,000） vs. リドカイン6㎎

## 6-2　投与群

リドカイン徐放シートの適切な投与量を決定するため投与群は3群設定する。

| 群 | 数 |
| --- | --- |
| A非投与群 | 20症例 |
| B対照群（PLGA100mg） | 20症例 |
| 投与群 | 60症例 |
| C（SRLS100mg） | 20症例 |
| D（SRLS200mg） | 20症例 |
| E（SRLS400mg） | 20症例 |

## 6-3　治療等の介入を行う手順と経時的なスケジュール

### 6-3-1　投与部位：

試験薬投与部位：　抜歯後の手術創部内（ソケット内）

### 6-3-2　治療等の介入を行う期間

試験薬投与期間は１週間とする。投与後１週間（抜糸時）の診察で残存する試験薬は除去する。

### 6-3-3　用法・用量

塩酸プロピトカイン(歯科用シタネスト・オクタプレシンカートリッジ®)で局所麻酔後、抜歯を開始し、局所麻酔は術中適宜追加できる。閉創時にリドカイン徐放シートをソケットに留置して縫合終了する。D（SRLS200mg）はC（SRLS100mg）を2枚、E（SRLS400mg）はC（SRLS100mg）を4枚とする。また、Aは今まで通り酸化セルロース（サージセル®）を、Bはコントロールとして薬効のない基材（PLGA100mg）のみを留置して縫合終了とする。

### 6-3-4　増量・減量の目安等

　予定なし

### 6-3-5　併用薬及び併用療法

①　併用禁止薬及び禁止療法

試験結果に影響するため、試験プロトコールに記載以外の鎮痛薬の使用は禁止する。また、リドカインの血中濃度に影響を及ぼすため以下の薬物の使用も禁止する。

| 薬剤名等 | 臨床症状 | 機序・危険因子 |
| --- | --- | --- |
| シメチジン | リドカインの血中濃度が上昇したとの報告がある。 | シメチジンの肝代謝酵素阻害作用により、リドカインの代謝が抑制されると考えられる。 |
| メトプロロール  プロプラノロール  ナドロール | リドカインの血中濃度が上昇することがある。 | これらの薬剤の心拍出量、肝血流量減少作用により、リドカインの代謝が遅延すると考えられる。 |
| リトナビル  アンプレナビル  ホスアンプレナビルカル  シウム水和物  硫酸アタザナビル | リドカインのAUCが上昇することが予想される。 | 肝代謝酵素に対する競合的阻害作用により、リドカインの代謝が遅延すると考えられる。 |
| セイヨウオトギリソウ  （St.　John's　Wort,セン  ト・ジョーンズ・ワート）含  有食品 | リドカインの代謝が促進され血中濃度が低下するおそれがある。 | 肝代謝酵素誘導作用により、リドカインの代謝が促進され、血中濃度が低下すると考えられる。 |
| クラスIII抗不整脈剤  アミオダロン等 | 心機能抑制作用が増強するおそれがある。 | 併用により血中濃度が上昇し、作用が増強することが考えられる。 |

②　併用可能薬・可能療法

術後、鎮痛薬レスキューとしてセレコキシブ（セレコックス®）を初回のみ４００ｍｇ、必要に応じて以降は２００ｍｇを６時間以上あけて１日２回まで経口投与できる。さらに、セレコキシブ投与後２時間以上たっても効果が不十分で、次の６時間後のセレコキシブまで待てない場合に限りアセトアミノフェン（カロナール®）１回４００ｍｇも追加経口投与し、必要に応じて２時間以上あけて１日４０００ｍｇ（１０回）まで追加経口投与可能とする。したがって、優先順位はセレコキシブでありセレコキシブ投与から６時間以上あいている場合は、まずセレコキシブを優先し投与し、それでも効果不十分の場合、アセトアミノフェンを追加投与することになる。

また術後、抗生物質としてセフカペンピボキシル塩酸塩水和物（フロモックス®）３００ｍｇ分３を３日間経口投与する。

# **（７）観察・検査・調査項目及び実施時期**

## 7-1　観察・検査項目

VAS

鎮痛薬レスキュー：指定した鎮痛薬の使用量を調査する。

患者満足度

血液検査：血球数、ヘモグロビン、血小板数、総タンパク、AST、ALT、Na、K、Cl

尿素窒素、クレアチニン、血中リドカイン濃度

有害事象：適応創部の状態を含めすべての有害事象を調査する。必要であれば診察を行い自他覚所見も調査する。

12誘導心電図検査

## 7-2　観察・検査方法

症例報告書^7）^を使用し研究担当医師及び患者が記入する。

## 7-3　実施時期・スケジュール

|  | 前 | 開始 | 4H | 8H | 24H | 2D | ～ | 6D | 7D | 2W |
| --- | --- | --- | --- | --- | --- | --- | --- | --- | --- | --- |
| 同意書 | ○ |  |  |  |  |  |  |  |  |  |
| 登録 |  | ○ |  |  |  |  |  |  |  |  |
| VAS |  |  | ○ | ○ | ○ | ○ | ○ | ○ | ○ |  |
| 鎮痛薬レスキュー |  |  |  |  | ○ | ○ | ○ | ○ | ○ |  |
| 患者満足度 |  |  |  |  | ○ | ○ | ○ | ○ | ○ |  |
| 有害事象・診察 | ○ |  |  |  | ○ | 必要に応じ受診 | | | ○ | ○ |
| 血中リドカイン濃度 | ○ |  |  |  | ○ |  |  |  | ○ |  |
| 血液検査 | ○ |  |  |  |  |  |  |  | ○ | ○ |
| 12誘導心電図 | ○ |  |  |  |  |  |  |  |  |  |
| 症例報告書 | 作成 |  |  |  |  |  |  |  |  | 提出 |

# **（８）重篤な有害事象への対応**

重篤な有害事象及び予測できない新たな有害事象が発現した場合、臨床研究責任医師又は臨床研究分担医師は適切な処置を行うとともに、研究実施期間中はすべての有害事象（自他覚症状や検査値異常等）について内容・発現時期・消失時期・程度・処置・転帰・重篤性評価を記録し、試験薬との関連性をカルテに記載する。必要に応じて追跡調査も行う。

# **（９）研究の中止基準**

## 9-1　患者（被験者）ごとの中止基準

以下のような場合には試験を中止する。試験を中止した場合は、その理由を明らかにして、カルテに記載する。

1. 被験者より同意の撤回があった場合
2. リドカイン中毒の疑いがあった場合
3. 追加治療を必要とする創部感染をした場合
4. 試験薬の成分に対しアレルギー反応が出た場合
5. 試験プロトコール以外の鎮痛薬の使用など、併用禁止薬を使用した場合
6. 妊娠していることがわかった場合
7. 患者が来院しない場合
8. その他重篤な有害事象が発生した場合
9. その他主治医が試験続行困難と判断した場合

## 9-2　研究全体の中止基準

下記に該当した場合は試験全体を中止する。臨床研究責任医師は、試験を中止する場合には被験者に中止することを速やかに通知し、適切な医療の提供やその他の必要な措置を講ずる。

　　　　　　1)臨床研究実施医療機関のIRBが試験を継続すべきでないと判断した場合

　　　　　　2)試験の安全性に疑義が生じた場合

# **（１０）患者（被験者）の登録方法・割付方法**

当施設を割り付けセンターとし各施設の症例を5群へランダムに割り付ける。コンピュータを使ってA～Eまでのボールを10ずつ50個箱に入れ、1つずつ引いて割り付けを行いランダム化する。引いたボールは元に戻さず20症例に割り付けたらすべて元に戻しこれを2回繰り返す。担当医師はその都度、電子メールで問い合わせを行って割り付けを確認し登録する。

# **（１１）研究実施期間**

2014年1月～2017年3月（症例登録期間は2016年12月まで）

# **（１２）予定症例数**

非投与群、対照群、各投与群はそれぞれ20症例とする。

先行研究^2）^の結果から痛み閾値のAUCを見積もると、2群間の平均値の差: 104, 2群共通の標準偏差: 132, αエラー: 0.05, 解析方法: 両側, 検出力（1-β）: 0.8とすると予定症例数は15となる。脱落症例も考慮し各群を20症例とした。

# **（１３）統計学的事項**

13-1　有効性評価項目

13-1-1　主要評価項目（プライマリーエンドポイント）

VAS値

鎮痛薬レスキュー回数

患者満足度

上記評価項目を対照群と比較する。

13-1-2　副次的評価項目（セカンダリーエンドポイント）

VAS値

鎮痛薬レスキュー回数

患者満足度

上記評価項目を非投与群と比較する。

13-2　安全性評価項目

有害事象

血液検査（血球算定、生化学）

血中リドカイン濃度

13-3　解析方法

AUCの解析はone-way ANOVA検定の後、Tukey法をつかったpost-hoc検定を行う。経時的変化の解析にはTwo- way repeated measure ANOVA検定の後、Dunnett法を使ったpost-hoc検定を行う。必要に応じFull analysis /Per protocol analysis/As-treated analysisを行う予定である。

13-4　中間解析と研究の早期中止

　予定なし。

# **（１４）臨床試験審査委員会への報告義務**

①　重篤な有害事象が発生した場合

②　プロトコールの変更を行う場合

③　終了若しくは中止する場合

④　臨床研究責任医師、臨床研究分担医師の変更を行う場合

⑤ その他

# **（１５）症例報告書の取り扱い**

症例報告書^7）^は紛失や漏えいを防ぐため自ら現地へ収集に行き、郵送・電子メール等の媒体は用いない。

# **（１６）倫理的事項**

16-1　遵守すべき諸規則

GCP、ヘルシンキ宣言、臨床研究に関する倫理指針を遵守する。

16-2　インフォームド・コンセントの手順

臨床研究責任医師、臨床研究分担医師は被験者に対して別に定める説明・同意文書に基づき、本研究に参加する前に研究の内容について十分に説明する。研究に参加するかどうかについて十分考える時間を与えた後、臨床研究責任、臨床研究分担医師は本人の自由意思による研究参加の同意を文書（別途定める同意文書）で得る。また、国外共同研究実施医療機関で行う場合は現地研究担当者により現地言語により、上記の通り十分に説明し同意を文書で得る。

16-3　同意説明文書の内容

　　研究責任医師は、説明文書・同意書を作成する。また作成した説明文書・同意書は試験開始前に臨床試験審査委員会に提出し、その承認を得る。説明文書には、少なくとも「臨床研究に関する倫理指針」に定められた事項について記載しなければならない。ただし、被験者を意図的に誘導するような記載をしてはならない。

1. 臨床研究は治療以外に研究を伴うこと
2. 臨床研究の目的
3. 臨床研究の方法
4. 被験者の臨床研究への参加予定期間
5. 臨床研究に参加する予定の被験者数
6. 予期される臨床上の利益及び危険性又は不便
7. 当該疾患に対する他の治療方法について
8. 臨床研究に参加した場合の費用と健康被害を受けた場合の治療及び補償
9. 臨床研究への参加は被験者の自由意思によるもので、臨床研究への参加を随時拒否・撤回できること。また、これによって被験者が不利な扱いを受けないこと
10. 臨床研究の継続について被験者の意思に影響を与える可能性のある情報が得られた場合には速やかに被験者に伝えられること
11. 臨床研究への参加を中止させる場合の条件又は理由
12. 被験者の秘密が保全されることを条件に、モニター、監査担当者、臨床試験審査委員会及び規制当局が医療記録等を閲覧出来ること。その際、被験者の秘密は保全されること。また、同意文書に被験者が記名捺印又は署名することによって閲覧を認めたことになること
13. 臨床研究の結果が公表される場合であっても、被験者の秘密は保全されること
14. 臨床研究に係る資金源、起こりうる利害の衝突
15. 臨床研究責任医師の氏名・職名・連絡先
16. 当該臨床研究に関する照会連絡先
17. 被験者が守るべき事項

16-4 研究内容の公開

研究の実施についてホームページ等で公開する予定なし。

16-5 患者（被験者）の個人情報及びプライバシーの保護

　　被験者のプライバシー保護のため、セキュリティーを保ったコンピュータ環境の中で処理、保存が行われ、個々の被験者の識別には被験者識別コードを用い、被験者の個人情報を保護する。また、個人情報保護のため症例報告書を含む試験結果の情報は自ら現地へ収集に行き、郵送・電子メール等の媒体は用いない。

# **（１７）健康被害に対する補償・賠償**

　　当該共同研究実施医療機関の制度に従うものとするが、群馬大学医学部附属病院においては本研究の参加または終了後に本研究に参加したことが原因となって、重篤な副作用などの健康被害を受けた場合には、通常の診療と同様に適切に治療を行う。その際の医療費は通常の保険診療にて賄う。また、本研究の試験薬（薬効のない基材またはSRLS）に起因して後遺障害が生じた場合はその程度に応じて補償・賠償金を支払い、臨床研究に関わる責任保険にて補填する。

# **（１８）予測される医療費（患者（被験者）の負担）**

本研究に参加した場合、健康保険が適用になっていない研究に伴う検査（血液検査、心電図検査等）にかかる費用については病院が負担するため、患者の負担となることはない。しかし、患者の病気の治療にかかる医療費のうち健康保険からの給付を除く部分は通常の診療同様、自己負担になる。本研究に参加することで患者（被験者）の費用負担は増えないが、逆に試験薬の効果により負担が減ることも考えられる。

# **（１９）患者（被験者）に対する金銭の支払、医療費の補助**

なし。

**（２０）研究資金の拠出元**

本研究に要する費用は、臨床研究責任医師を研究代表者とする独立行政法人日本学術振興会の科学研究費をもって充てる。

**（２１）利益相反**

本研究の利害関係については、群馬大学利益相反マネジメント委員会の承認を得て行なう。また、当該研究経過を定期的に群馬大学利益相反マネジメント委員会へ報告等を行うことにより、本研究の利害関係についての公平性を保つ。

**（２２）研究実施計画書の改訂**

　研究代表者は、本研究開始後に研究実施計画書の改訂が必要になった場合、軽微な場合を除き、改訂の場合には、臨床試験審査委員会で改訂内容とその理由等について、再度審査を受け、承認を得る。

**（２３）研究に関する資料等の利用と保存**

症例報告書は臨床研究責任医師が確認後、麻酔神経科学教室で保存し、そのデータはセキュリティーを保ったコンピュータ環境の中で処理し保存する。記録（文書及び電子記録）の保存期間は、研究終了日から3年間とする。保存責任者は臨床研究責任者：鈴木敏之である。

**（２４）特記事項**

なし。

**（２５）研究成果の帰属と結果の公表**

本研究の結果として、特許権等が生じる場合には、その権利は、群馬大学（研究者）に帰属し、試（資）料提供者には属さない。しかし、本研究で得られた研究成果はPadjadjaran Universityとの共同研究として学会発表、論文化等を行う予定である。

**（２６）研究組織及び連絡先**

臨床研究責任医師

群馬大学医学部附属病院麻酔科蘇生科　助教　鈴木敏之（院内PHS 25635）

〒371-8511

群馬県前橋市昭和町 3-39-15

TEL 027-220-8454（直通）

FAX 027-220-8473

国外共同研究実施医療機関

Department of Anesthesiology and Intensive Care

Hasan Sadikin Hospital / Medical Faculty, Padjadjaran University

Professor Ike Sri Redjeki

Professor Eri Surahman

Bandung, Indonesia

(+62) 22 2038285

**（２７）参考資料、文献リスト、付録**

1. Tobe M, Obata H, Suto T, Yokoo H, Nakazato Y, Tabata Y, Saito S.

Department of Anesthesiology, Gunma University Graduate School of Medicine, Maebashi, Japan.

Long-term effect of sciatic nerve block with slow-release lidocaine in a rat model of postoperative pain. Anesthesiology. 2010 Jun;112(6): 1473-81

1. 鈴木敏之1,戸部賢1,松岡宏晃1,須藤貴史1,小幡英章1,齋藤繁1（1.群馬大学医学部附属病院 麻酔科蘇生科）

リドカイン徐放シートのヒト正常粘膜における安全性と有効性を確認した第Ⅰ/Ⅱ相臨床試験

日本麻酔科学会第60回学術集会プログラム2013, vol. 62: 113 [Q13-1]

1. Golf M, Daniels SE, Onel E.

Premier Clinical Research Centers, Austin, TX, USA.

A phase 3, randomized, placebo-controlled trial of DepoFoam® bupivacaine (extended-release bupivacaine local analgesic) in bunionectomy.

Adv Ther. 2011 Sep; 28(9):776-88.

1. SAFETY DATA SHEET; LACTEL® Absorbable Polymers

Durect社

1. SAFETY DATA SHEET; Lidocaine（L7757）

SIGMA-ALDRICH社

1. SAFETY DATA SHEET; Chloroform（C2432）

SIGMA-ALDRICH社

1. 症例報告書

①担当医師用

②患者さん用

1. Protocol of the clinical study（英語版）
