# Supplementary material for: Sustained-release lidocaine sheet for pain following tooth extraction: A randomized, single-blind, dose-response, controlled, clinical study of efficacy and safety
Source: PLoS One. 2018 Jul 2;13(7):e0200059. doi: 10.1371/journal.pone.0200059 (PMC6028143; doi:10.1371/journal.pone.0200059)
Supplement: S2 Protocol — (DOC) [file pone.0200059.s003.doc]

# **Title Page**

Clinical trial protocol

Phase 1/2 clinical trial of a sustained-release lidocaine sheet for postoperative pain

Assistant Investigator

Kensuke Kosugi, D.D.S., Ph.D.

Department of Oral and Maxillofacial Surgery

Gunma University Graduate School of Medicine

Investigator

Toshiyuki Suzuki, MD., PhD.

Department of Anesthesiology

Gunma University Graduate School of Medicine

Chief Investigator

Shigeru Saito, MD., PhD

Department of Anesthesiology

Gunma University Graduate School of Medicine

Date of Creation: July 3, 2013 Version No. 1

July 30, 2013 Version No. 2

July 31, 2013 Version No. 2.1

August 20, 2013 Version No. 3

August 21, 2013 Version No. 4

October 29, 2013 Version No. 4.1

January 28, 2014 Version No. 4.2

March 24, 2014 Version No. 4.3

August 10, 2014 Version No. 4.4

**Table of Contents**

[**Title Page** 1](#__RefHeading___Toc496580919)

[**Definitions of Abbreviations and Terms** 4](#__RefHeading___Toc496580920)

[**(1) Background to the Study and Significance and Rationale of the Study** 5](#__RefHeading___Toc496580921)

[**(2) Objective of the Study** 5](#__RefHeading___Toc496580922)

[**(3) Standards and Definitions Used in the Study** 5](#__RefHeading___Toc496580923)

[**(4) Study Protocol / Study Design** 6](#__RefHeading___Toc496580924)

[4-1 Design 6](#__RefHeading___Toc496580925)

[4-2 Overall Study Design 6](#__RefHeading___Toc496580926)

[**(5) Patient (Volunteer) Selection Criteria** 6](#__RefHeading___Toc496580927)

[5-1 Inclusion criteria 7](#__RefHeading___Toc496580928)

[5-2 Exclusion criteria 7](#__RefHeading___Toc496580929)

[**(6) Specific Details of Study Intervention** 7](#__RefHeading___Toc496580930)

[6-1 Outline of Study Drug, Medical Device, and Biomedical Material 7](#__RefHeading___Toc496580931)

[6-2 Groups 8](#__RefHeading___Toc496580932)

[6-3 Intervention Procedures and Schedule 9](#__RefHeading___Toc496580933)

[6-3-1 Administration Site 9](#__RefHeading___Toc496580934)

[6-3-2 Intervention Period 9](#__RefHeading___Toc496580935)

[6-3-3 Dosage and Administration 9](#__RefHeading___Toc496580936)

[6-3-4 Standards for Dose Escalation and Dose Reduction 9](#__RefHeading___Toc496580937)

[6-3-5 Concomitant Drugs and Therapies 9](#__RefHeading___Toc496580938)

[**(7) Assessment Items and Timing of Assessments** 10](#__RefHeading___Toc496580939)

[7-1 Assessment Items 10](#__RefHeading___Toc496580940)

[7-2 Assessment Methods 10](#__RefHeading___Toc496580941)

[7-3 Implementation Timings and Schedule 10](#__RefHeading___Toc496580942)

[**(8) Dealing with Serious Adverse Events** 10](#__RefHeading___Toc496580943)

[**(9) Study Discontinuation and Withdrawal Criteria** 11](#__RefHeading___Toc496580944)

[9-1 Individual Patient (Volunteer) Withdrawal 11](#__RefHeading___Toc496580945)

[9-2 Overall Study Discontinuation 11](#__RefHeading___Toc496580946)

[**(10) Patient (Volunteer) Enrollment and Assignment** 11](#__RefHeading___Toc496580947)

[**(11) Study Period** 11](#__RefHeading___Toc496580948)

[**(12) Anticipated Numbers of Patients** 11](#__RefHeading___Toc496580949)

[**(13) Statistical Analysis** 12](#__RefHeading___Toc496580950)

[13-1 Efficacy Assessments 12](#__RefHeading___Toc496580951)

[13-1-1 Primary Endpoints 12](#__RefHeading___Toc496580952)

[13-1-2 Secondary Endpoints 12](#__RefHeading___Toc496580953)

[13-2 Safety Assessments 12](#__RefHeading___Toc496580954)

[13-3 Analysis Method 12](#__RefHeading___Toc496580955)

[13-4 Interim Analysis and Early Study Discontinuation 12](#__RefHeading___Toc496580956)

[**(14) Mandatory Reporting to Institutional Review Board** 12](#__RefHeading___Toc496580957)

[**(15) Handling of Case Report Forms** 12](#__RefHeading___Toc496580958)

[**(16) Ethical Considerations** 13](#__RefHeading___Toc496580959)

[16-1 Regulatory Compliance 13](#__RefHeading___Toc496580960)

[16-2 Informed Consent Procedure 13](#__RefHeading___Toc496580961)

[16-3 Content of Informed Consent Document 13](#__RefHeading___Toc496580962)

[16-4 Publicizing the Details of the Study 14](#__RefHeading___Toc496580963)

[16-5 Safeguarding Patient (Volunteer) Privacy and Individual Information 14](#__RefHeading___Toc496580964)

[**(17) Compensation and Reimbursement for Damage to Health** 14](#__RefHeading___Toc496580965)

[**(18) Anticipated Medical Expenses (to be Borne by the Patient or Volunteer)** 14](#__RefHeading___Toc496580966)

[**(19) Monetary Payments to Patients (Volunteers) and Assistance with Medical Expenses** 14](#__RefHeading___Toc496580967)

[**(20) Source of Funding for the Study** 14](#__RefHeading___Toc496580968)

[**(21) Conflict of Interest** 14](#__RefHeading___Toc496580969)

[**(22) Amendment of the Study Protocol** 14](#__RefHeading___Toc496580970)

[**(23) Usage and Storage of Study Documentation** 15](#__RefHeading___Toc496580971)

[**(24) Special Notices** 15](#__RefHeading___Toc496580972)

[**(25) Ownership and Publication of the Study Results** 15](#__RefHeading___Toc496580973)

[**(26) Study Organization and Contact Details** 15](#__RefHeading___Toc496580974)

[**(27) Reference Data, References, and Appendices** 15](#__RefHeading___Toc496580975)

**Definitions of Abbreviations and Terms**

The abbreviations and terms used in this study protocol are shown below.

------------------------------------------------------------------------------

Abbreviation

or Specialist Term Definition

------------------------------------------------------------------------------

ANOVA Analysis Of Variance

ALT Alanine Aminotransferase

AST Aspartate Aminotransferase

ASA American Society of Anesthesiologists

AUC Area Under the Curve

GCP Good Clinical Practice

IV Inherent Viscosity

NSAIDs Non-Steroidal Anti-Inflammatory Drugs

PLGA Poly (Lactic-co-Glycolic Acid)

PS Physical Status

SRLS Slow-Release Lidocaine Sheet

VAS Visual Analog Scale

------------------------------------------------------------------------------

**(1) Background to the Study and Significance and Rationale of the Study**

Postoperative pain relief is a subject of recently growing interest, and varied pain relief methods based on a range of analgesics are used depending on such factors as the nature of the operation and the risk to the patient. However, the current situation in the field of postoperative pain relief cannot be described as completely satisfactory. For example, operations on patients taking oral anticoagulants and anti-platelet agents have increased, cases of early postoperative initiation of anticoagulant therapy have also increased, and epidural anesthesia tends to be less frequently used due to the risk of hematoma-induced neuropathy. Continuous intravenous opioid infusion is one alternative being increasingly used; however, opioids have a weak effect on movement-related pain, and are associated with adverse reactions which prevent full pain relief such as nausea, vomiting, drowsiness, and respiratory depression. Ultrasonically guided peripheral nerve blocks have also seen a recent increase in their use as the resolution of the equipment improves. Such nerve blocks represent a safe and very satisfactory method of pain relief; however, the necessity for technology acquisition and the substantial early-phase costs are problematic. Long-acting pain relief requires continuous medication, whatever strategy is selected. Against this background, another approach concerns releasing local anesthetics in small amounts over a prolonged period (sustained release). When achievable, it is considered that such an approach will be effective even for movement-related pain, and will provide safe postoperative pain relief with few adverse reactions, through single administration to the site of the surgical wound and the surrounding dominant nerve. Slow-Release Lidocaine Sheet (SRLS) was developed using biodegradable material by Gunma University in 2010. The material, which is degraded and absorbed in the body and ultimately broken down into carbon dioxide and water, is already in clinical use as an absorbable suture. The safety and long-term action of SRLS when used with sciatic nerve blocks in rats were confirmed at the university in the same year^1)^. Subsequently, the safety and long-term action of SRLS when applied externally to healthy mucous membranes in human volunteers were confirmed in a clinical study approved by the Institutional Review Board (IRB) at Gunma University^2)^. Internationally, liposomal bupivacaine is the only local anesthetic which has reached clinical use as a sustained-release agent^3)^. SRLS is a differently formulated product, and its clinical use has not been the subject of any international report. Such a formulation could provide effective analgesia for a few days to a number of weeks. Accordingly, any research leading to its development would be very highly original. Pain relief has great significance for society, and it is considered that the development of an effective sustained-release local anesthetic which elicits few adverse reactions would represent a great contribution to health economics as well as medical progress.

**(2) Objective of the Study**

The objective of this study is to investigate the safety (doses and the toxic dose) and efficacy of SRLS for post-extraction pain when applied to patients after tooth extraction.

**(3) Standards and Definitions Used in the Study**

Pain will be assessed using a visual analog scale (VAS) scale. On this scale, “0-mm” represents “no pain” and 100-mm represents “I have never experienced such pain before (“The strongest pain I have ever experienced”), and respondents will be asked to rate their current level of pain by indicating a point on the 100-mm long straight line (connecting the points for 0-mm and 100-mm), and these indications will be used for the assessment. This method is the most common such method used in medical care.

Patient satisfaction will be assessed using a five-step scale on which “1” represents “dissatisfied”, “2” represents “rather dissatisfied”, “3” represents “neither”, “4” represents “rather satisfied”, and “5” represents “satisfied”.

The American Society of Anesthesiologists physical status (ASA PS) system will be used to classify the surgical patients into six grades by their general health; the preoperative classification is regarded as being associated with patient prognosis.

ASA PS 1 - A normal healthy patient

ASA PS 2 - A patient with mild systemic disease

ASA PS 3 - A patient with severe systemic disease

ASA PS 4 - A patient with severe systemic disease that is a constant threat to life

ASA PS 5 - A moribund patient who is not expected to survive without the operation

ASA PS 6 - A declared brain-dead patient whose organs are being removed for donor purposes

# **(4) Study Protocol / Study Design**

## 4-1 Design

Phase 1/2 clinical trial (Single-blind randomized trial)

## 4-2 Overall Study Design

| Obtaining written informed consent |
| --- |

| Screening prior to the start |
| --- |

| Enrollment |
| --- |

| Randomization |
| --- |

| Non – administration (A) | Control (B) | Administration (C~E) |
| --- | --- | --- |

| Checking at the end |
| --- |

Joint research

| **Gunma University** | **Padjadjaran University** |
| --- | --- |

| Production of the study drugs |
| --- |

approved by the IRB of Padjadjaran University Hospital

| Clinical trial | Clinical trial |
| --- | --- |

| Data analysis |
| --- |

| Publication of a paper on the study |
| --- |

Authorship for Padjadjaran members & Gunma members

# **(5) Patient (Volunteer) Selection Criteria**

Patients who fulfill all the inclusion criteria stated below and do not fulfill any of the exclusion criteria stated below will be eligible for the study.

## 5-1 Inclusion criteria

1. 20 to 50 years of age
2. Male or female outpatient
3. Elective operation
4. ASA-PS 1, 2
5. Extraction of horizontally impacted mandibular wisdom tooth
6. Provided written consent to participate in this study

## 5-2 Exclusion criteria

1. A person taking a drug (e.g. antipsychotic, NSAIDs, or opioid) that is considered to affect pain relief or is judged to have a possible carry-over effect
2. A patient with notable surgical site infection
3. A patient who has an allergy to Lidocaine, an amide type local anesthetic agent, Celecoxib, a sulfonamide, or Acetaminophen
4. A patient with a serious disturbance of the cardiac conduction system (e.g. complete atrioventricular block)
5. A patient with peptic ulcer or asthma
6. A patient who has disturbed consciousness or is unable to communicate effectively for evaluation
7. A patient who is judged by the attending physician to be unsuitable for this trial

# **(6) Specific Details of Study Intervention**

6-1 Outline of Study Drug, Medical Device, and Biomedical Material

Study Drug： 40% SRLS

Material： PLGA (50:50), IV 0.55-0.75; DURECT, LACTEL® Absorbable Polymers,

50:50 Poly (DL-lactide-co-glycolide) Ester Terminated Polymers,

Inherent Viscosity 0.55-0.75, Product No: B6010-2^4）^

Lidocaine (with no hydrochloride);

SIGMA-ALDRICH, lidocaine powder, Product No: L7757^5）^

Method of Preparation:

1. The material will be placed into a vial and dissolved with chloroform^6)^.
2. After confirmation of dissolution, the solution will be spread on petri dishes on a level bench.
3. The petri dishes will be not covered by lids and will be placed in a safety cabinet for two days at ambient temperature (around 25˚C) to evaporate the solvent. The safety cabinet will be irradiated with a germicidal lamp for at least two hours before use.
4. The petri dishes will be enclosed under lids and placed in a vacuum drying apparatus for 14 days at around 37˚C to 40˚C to further evaporate solvent.
5. Gamma ray radiation will be applied
6. The resultant formulation will be stored frozen (−20˚C or below).

Ingredients: 40 mg lidocaine per 100 mg study drug (≤10 mg chloroform)

Sustained Release Capability: Mean±standard deviation (SD) (n=4) value used for the in vitro sustained release curve [40% SRLS (50:50, PLGA, IV 0.55-0.75)]

Hemodynamics^1)^: Mean±SD serum lidocaine concentrations in Sprague-Dawley rats (250 g to 300 g; n=6 per group). 20 mg 30% SRLS (6 mg lidocaine; 50:50, PLGA, molecular weight 85,000) vs. 6 mg lidocaine.

## 6-2 Groups

Three dose groups will be established to determine the appropriate dose of SRLS.

| Groups | Interventions | Sample size |
| --- | --- | --- |
| Non– administration (A) | none | 20 |
| Control (B) | PLGA 100mg | 20 |
| Administration (C) | SRLS 100mg | 20 |
| Administration (D) | SRLS 200mg | 20 |
| Administration (E) | SRLS 400mg | 20 |

## 6-3 Intervention Procedures and Schedule

### 6-3-1 Administration Site

Study Drug Administration Site: Within the post-tooth extraction surgical wound (within the socket)

### 6-3-2 Intervention Period

The study drug administration period will be set at one week. Any remaining study drug will be removed at the examination after one-week administration (at the time of suture removal).

### 6-3-3 Dosage and Administration

A dentist will start tooth extraction under local anesthesia with propitocaine hydrochloride and felypressin can be added as appropriate during the operation. After the extraction, a SRLS containing the study drug is inserted into the socket and the socket is close by surgical sutures. The Non-administration group will receive the conventional extraction; the PLGA 100 mg control group will receive the PLGA matrix without lidocaine; the SRLS 100 mg group will receive a single sheet of SRLS 100 mg; the SRLS 200 mg group will receive double sheets of SRLS 100 mg; and the SRLS 400 mg administration group will receive four sheets of SRLS 100 mg. The administration period will be two weeks. In other words, the remaining study drug will be removed when checking the condition at one week after suture removal.

### 6-3-4 Standards for Dose Escalation and Dose Reduction

Not planned

### 6-3-5 Concomitant Drugs and Therapies

1. Prohibited concomitant drugs and therapies

The use of any analgesics not stated in the study protocol is prohibited, due to the potential effect on the study results. The use of any of the below-stated drugs is also prohibited due to the potential effect on blood lidocaine concentration measurement.

| Drugs | Clinical reaction/effect (potential) | Mechanism/Risk factor |
| --- | --- | --- |
| Cimetidine | Blood lidocaine concentration is reported to increase. | Lidocaine metabolism is considered to be suppressed as a result of the inhibition of hepatic metabolizing enzymes by cimetidine. |
| Metoprolol  Propranolol  Nadolol | Blood lidocaine concentration increases in some cases. | Lidocaine metabolism is considered to be delayed as a result of the decreases in cardiac output and hepatic blood flow induced by these drugs. |
| Ritonavir  Amprenavir  fosamprenavir  atazanavir | Lidocaine AUC is predicted to increase. | Lidocaine metabolism is considered to be delayed as a result of the competitive inhibition of hepatic metabolizing enzymes. |
| St.　John's　Wort  (hypericum perforatum) | There is a risk of decreased blood lidocaine concentration after acceleration of its metabolism. | Blood lidocaine concentration is considered to decrease after its metabolism is accelerated as a result of induction of hepatic metabolizing enzymes. |
| Class III antiarrhythmic agents  (e.g. Amiodarone) | There is a risk of amplification of the suppressive effect on cardiac function. | The blood concentration is considered to be increased and the drug effect amplified as a result of concomitant use. |

1. Permitted concomitant drugs and therapies

A patient may take oral celecoxib (Celecox ®) 200 mg (400 mg the first time) as analgesic rescue postoperatively and can repeat it after an interval of more than six hours to a maximum of twice a day, as needed. However, if the pain relief is insufficient and the patient cannot wait until the next celecoxib dose, the patient may take oral acetaminophen (Calonal ®) 200 mg more than two hours after taking celecoxib, and can repeat it after an interval of more than two hours to a maximum of 4,000 mg (20 times) a day, as needed. Therefore, the priority is celecoxib. If it is taken more than six hours from the last administration of celecoxib, preference should be given to celecoxib, and more acetaminophen should be taken two hours later in the case the pain relief is insufficient.

In addition, patients will take oral cefcapene pivoxil hydrochloride hydrates (Flomox ®) 300 mg a day as postoperatively routine antibiotics for three days.

# **(7) Assessment Items and Timing of Assessments**

## 7-1 Assessment Items

VAS

Analgesic rescue: To be investigated at the stipulated analgesic dose

Patient satisfaction

Blood testing:

Blood cell counts, hemoglobin, platelet count, total protein, AST, ALT, sodium, potassium, chlorine, urea nitrogen, creatinine, and blood lidocaine concentration

Adverse events:

All adverse events, including those concerning the state of the surgery site.

Patients will be examined and subjective and objective symptoms will be investigated as required.

12-lead electrocardiography

## 7-2 Assessment Methods

Case Report Forms^7)^ will be used, and completed by the investigator and the patient.

## 7-3 Implementation Timings and Schedule

|  | Pre- | Start | 4 h | 8 h | 24 h | 2 d to 6 d | 7 d | 14 d |
| --- | --- | --- | --- | --- | --- | --- | --- | --- |
| Informed consent | 〇 |  |  |  |  |  |  |  |
| (Enrollment)Tooth extraction |  | ○ |  |  |  |  |  |  |
| VAS |  |  | ○ | ○ | ○ | ○ | ○ |  |
| Painkiller rescue |  |  | ○ | ○ | ○ | ○ | ○ |  |
| Satisfaction |  |  |  |  | ○ | ○ | ○ |  |
| Hospital visit | 〇 | ○ |  |  | ○ | As needed | ○ | ○ |
| Lidocaine concentration | 〇 |  |  |  | ○ |  | ○ |  |
| Blood examinations | 〇 |  |  |  |  |  |  | ○ |
| ECG | 〇 |  |  |  |  |  |  |  |
| Questionnaire sheets | prepared |  |  |  |  |  |  | collected |

# **(8) Dealing with Serious Adverse Events**

When serious adverse events and unexpected adverse events occur, the investigator or other physician in attendance will deal with them appropriately. For all adverse events (subjective or objective symptoms, clinical laboratory test abnormalities, etc.) occurring during the study period, the investigator or other physician in attendance will record the details, time of expression, time of resolution, grade, treatment measures, outcome, and evaluation of seriousness. The relationship to the study drug will be recorded in the relevant patient record. The patient may undergo additional examinations as required.

# **(9) Study Discontinuation and Withdrawal Criteria**

## 9-1 Individual Patient (Volunteer) Withdrawal

Patients will be withdrawn from the study in the event of any of the circumstances stated below. In the event a patient is withdrawn from the study, the reason for the withdrawal will be elaborated in the relevant patient record.

1. Withdrawal of consent
2. Suspicion of an addiction to lidocaine
3. Severe wound infection requiring additional treatment
4. Allergic reaction to the study drugs
5. Use of analgesics other than in accordance with the study protocol or use of prohibited drugs
6. Detection of pregnancy
7. Lost to follow-up
8. Occurrence of other serious adverse events
9. Judgment of an attending physician that it is difficult to continue to participate in this study

## 9-2 Overall Study Discontinuation

The study will be discontinued in the event of either of the circumstances below. If the study is discontinued, the investigator will promptly notify the patients, offer appropriate treatment, and take any other measures that may be required.

1. Judgment of the IRB at the trial site that this study should not be continued
2. Doubts regarding the safety of the study

# **(10) Patient (Volunteer) Enrollment and Assignment**

Gunma University will be regarded as the assignment center and the patients recruited at each center will be randomized into five groups. A computer will be used for the randomization, in which balls labeled A to E will be introduced in groups of 10, and placed in 50 individual slots, and balls will be drawn one by one for assignment. Individual balls will not be returned to the original container after being drawn. The balls will be used to assign 50 patients without being returned to their container, and after assigning 50 patients the balls will be returned. This procedure will be repeated two times. The investigator will verify assignments by e-mail inquiry for each ball-drawing session, and record them.

# **(11) Study Period**

January 2014 to March 2017 (Patients will be enrolled before December 2016.)

# **(12) Anticipated Numbers of Patients**

Patients will be assigned at 20 patients per group to the non-administration and control groups, and each administration group.

Based on the estimated area under the curve (AUC) for pain threshold for the results of a previous study^2)^, a total of 15 patients is anticipated to yield a power of test (1−β) of 0.8 in two-sided statistical analysis with an type 1 error rate of 0.05, a common standard deviation for two groups of 132, and difference of 104 between two group means. The number of patients per group has been set at 20 in consideration of possible patient drop-out.

# **(13) Statistical Analysis**

13-1 Efficacy Assessments

13-1-1 Primary Endpoints

The primary endpoint analysis will be performed by comparisons with the control group for the parameters stated below.

VAS score

Number of analgesic rescues

Patient satisfaction

13-1-2 Secondary Endpoints

The secondary endpoint analysis will be performed by comparisons with the non-administration group for the parameters stated below.

VAS score

Number of analgesic rescues

Patient satisfaction

13-2 Safety Assessments

Adverse events

Blood tests (blood cell counts and biochemistry)

Blood lidocaine concentration

13-3 Analysis Method

AUC data will be analyzed in statistical tests with one-way ANOVA, and then subject to post-hoc analysis with Tukey’s test. Chronological changes will be analyzed in statistical tests with two-way repeated measure ANOVA, and then subject to post-hoc analysis with Dunnett’s test. It is planned to perform full data set analysis, per-protocol analysis, and as-treated analysis as required.

13-4 Interim Analysis and Early Study Discontinuation

Not planned

# **(14) Mandatory Reporting to Institutional Review Board**

① Serious adverse event

1. Amendments to the protocol
2. Study completion or discontinuation
3. Change of investigator or other physician in attendance
4. Other cases

# **(15) Handling of Case Report Forms**

Case report forms^7)^ will be collected on-site, without using the postal service electronic mail, or other such forms of transmission, to protect against loss and disclosure.

# **(16) Ethical Considerations**

16-1 Regulatory Compliance

The study will be conducted in compliance with the relevant Good Clinical Practice (GCP) regulations, and in accordance with the Helsinki Declaration and Ethical Guidelines for Clinical Research.

16-2 Informed Consent Procedure

The investigator or other physician in attendance will fully explain the details of this study to the patients before they participate in the study based on a separately established Informed Consent Form. Prospective participants will be allowed sufficient time to consider whether they wish to enroll in the study or not, after which the investigator or physician in attendance will obtain their freely given written consent (separately established Informed Consent Form) to join the study. For the non-Japanese center collaborating in the study, the site investigator will provide an explanation and obtain informed consent in the manner outlined above, using the language local to that center.

16-3 Content of Informed Consent Document

The investigator will prepare the Informed Consent Form. This Informed Consent Form will be submitted to the IRB for approval before the start of the study. As a minimum, the items specified in the Ethical Guidelines for Clinical Research must be included in the Informed Consent Form; however, statements aimed at inducing patients’ voluntary consent may not be included.

1. The study involves research rather than treatment.
2. The objective of the study
3. The methodology of the study
4. The anticipated period of patient participation in the study
5. The anticipated number of patients who will participate in the study
6. Anticipated clinical benefits and risks or inconveniences
7. Other treatment options for relevant patients
8. Treatment and compensation for any damage to health or costs incurred while participating in the study
9. Participation in the study is voluntary; therefore, patients can refuse their participation or withdraw their consent at any time. Furthermore, patients will not be treated unfavorably in the event of such refusal or withdrawal.
10. If any information that might affect a patient’s intent to continue participating in the study is obtained, that information shall promptly communicated to patients.
11. Conditions or reasons for a patient’s participation in the study to be terminated
12. Monitors, auditors, the IRB, and regulatory authorities may view medical records under conditions of maintaining patient confidentiality. In affixing their seal or signing the Informed Consent Form, patients accept that their records may be viewed.
13. Patient confidentiality shall be maintained even in the event that the results of the study are published.
14. The sources of funding for the study and any possible conflicts of interest
15. The names, occupations, and contact details of the investigator
16. The contact details for making inquiries about this study
17. Rules that patients should observe

16-4 Publicizing the Details of the Study

It is not planned to make aspects of the conduct of the study public, such as by posting on the participating institutions’ web sites.

16-5 Safeguarding Patient (Volunteer) Privacy and Individual Information

Data will be processed and stored in secure computer environments to safeguard patient privacy. Individual patients are identified with Patient Identification Codes, to protect individual patient information. Furthermore, all information on study results including Case Report Forms will be collected on-site without using the postal service, or electronic mail, or other such forms of transmission, to safeguard individual information.

# **(17) Compensation and Reimbursement for Damage to Health**

Compensation and reimbursement will accord with the system at each center participating in the study; however, in the event that health damage such as a severe drug reaction resulting from study participation occurs during or after the end of this study at Gunma University Hospital, treatment will be provided in the same manner as regular medical care. The medical treatment costs at this time will be covered by normal health insurance. Furthermore, compensation and reimbursement will be paid for any physical impairment due to the study drug (SRLS or the non-effective base material) in this study in accordance with the extent of the impairment, and compensation will be received from the liability insurance for the study.

# **(18) Anticipated Medical Expenses (to be Borne by the Patient or Volunteer)**

Patients who participate in this study will not bear the cost of procedures in this study not covered by health insurance (such as blood tests and electrocardiography) because these costs will be borne by the center. However, the portion of a patients disease treatment costs that are not covered by health insurance will be borne by the patient in the same way as with normal medical care. Participation in the study must not impose any additional financial burden on the patient; rather, it is considered that the effect of the study drug will lead to a reduction of the burden.

# **(19) Monetary Payments to Patients (Volunteers) and Assistance with Medical Expenses**

None

**(20) Source of Funding for the Study**

As the Principal Investigator, the investigator will hold funding from Grant-in-Aid of Scientific Research made by Japan Society for the Promotion of Science for the costs necessitated by this study.

**(21) Conflict of Interest**

This study will be conducted after approval is obtained from the Conflict-of-Interest Management Committee of Gunma University with regard to conflicts of interest. Regular reports will also be made to this committee during the progress of this study, and the impartiality of this study will thus be assured.

**(22) Amendment of the Study Protocol**

In the event that the protocol of this study requires amendment after the initiation of the study, except in the event of minor changes, the Principal Investigator will present the details of, and the reason for, the amendment for further review by the IRB to obtain approval for the amendment.

**(23) Usage and Storage of Study Documentation**

The Case Report Forms will be checked by the investigator and then stored by the Department of Anesthesiology, Gunma University Graduate School of Medicine, and the data they contain will be processed and stored in a secure computer environment. Electronic and written records will be stored for a period of three years from the completion of the study. The investigator, Toshiyuki Suzuki, will be responsible for storage.

**(24) Special Notices**

None

**(25) Ownership and Publication of the Study Results**

Any patent rights developed as a result of this study and their privileges shall belong to Gunma University (researchers) and shall not belong to providers of samples (or data). However, it is planned that the results of this study will be presented at scientific meetings and published as collaborative research with Padjadjaran University.

**(26) Study Organization and Contact Details**

Investigator

Toshiyuki Suzuki, MD., PhD.

Department of Anesthesiology

Gunma University Graduate School of Medicine

3-39-22 Showa-machi, Maebashi-shi, Gunma371-8511, Japan

Phone: +81-27-220-8454 (Extension number 25635）

Fax: +81-27-220-8473

Joint Research Medical Institution

Professor Ike Sri Redjeki

Professor Eri Surahman

Department of Anesthesiology and Intensive Care

Hasan Sadikin Hospital / Medical Faculty, Padjadjaran University

Bandung, Indonesia

(+62) 22 2038285

**(27）Reference Data, References, and Appendices**

1. Tobe M, Obata H, Suto T, Yokoo H, Nakazato Y, Tabata Y, Saito S.

Department of Anesthesiology, Gunma University Graduate School of Medicine, Maebashi, Japan.

Long-term effect of sciatic nerve block with slow-release lidocaine in a rat model of postoperative pain. Anesthesiology. 2010 Jun;112(6): 1473-81

1. 鈴木敏之1,戸部賢1,松岡宏晃1,須藤貴史1,小幡英章1,齋藤繁1（1.群馬大学医学部附属病院 麻酔科蘇生科）

リドカイン徐放シートのヒト正常粘膜における安全性と有効性を確認した第Ⅰ/Ⅱ相臨床試験

日本麻酔科学会第60回学術集会プログラム2013, vol. 62: 113 [Q13-1]

1. Golf M, Daniels SE, Onel E.

Premier Clinical Research Centers, Austin, TX, USA.

A phase 3, randomized, placebo-controlled trial of DepoFoam® bupivacaine (extended-release bupivacaine local analgesic) in bunionectomy.

Adv Ther. 2011 Sep; 28(9):776-88.

1. SAFETY DATA SHEET; LACTEL® Absorbable Polymers

Durect社

1. SAFETY DATA SHEET; Lidocaine（L7757）

SIGMA-ALDRICH社

1. SAFETY DATA SHEET; Chloroform（C2432）

SIGMA-ALDRICH社

1. ①Case reports for a doctor

②Questionnaire sheets for a patient

1. Clinical trial Protocol（English）
