# Supplementary material for: Sustained-release lidocaine sheet for pain following tooth extraction: A randomized, single-blind, dose-response, controlled, clinical study of efficacy and safety
Source: PLoS One. 2018 Jul 2;13(7):e0200059. doi: 10.1371/journal.pone.0200059 (PMC6028143; doi:10.1371/journal.pone.0200059)
Supplement: S1 Table — (DOCX) [file pone.0200059.s004.docx]

|  | |  | Groups | | | | |  |  |  |
| --- | --- | --- | --- | --- | --- | --- | --- | --- | --- | --- |
|  | |  | None | PLGA  100mg | SRLS  100mg | SRLS  200mg | SRLS  400mg |  | Repeated Measures ANOVA |  |
|  |  |  |  |  |  |  |  |  |  |  |
| Time point | |  | N=20 | N=17 | N=20 | N=18 | N=19 |  |  |  |
| VAS scores  (Mean, SD) | 4 h |  | 33.8, 21.4 | 43.1, 31.4 | 35.7, 27.2 | 43.2, 28.9 | 26.6, 23.3 |  | P=0.1348 |  |
|  | 8 h |  | 26.1, 23.8 | 32.6, 20.3 | 23.2, 21.2 | 28.7, 27.3 | 25.2, 23.5 |  |  |  |
|  | 24 h |  | 14.0, 11.4 | 21.2, 25.0 | 10.1, 13.9 | 17.4, 18.2 | 14.1, 17.3 |  |  |  |
|  | 2 d |  | 13.3, 14.4 | 20.3, 25.6 | 11.7, 14.6 | 19.4, 21.2 | 9.4, 11.2 |  |  |  |
|  | 3 d |  | 14.3, 16.2 | 18.6, 23.4 | 7.2, 9.6 | 14.9, 11.9 | 7.6, 8.9 |  |  |  |
|  | 4 d |  | 15.8, 21.3 | 16.2, 19.0 | 6.5, 8.5 | 11.0, 10.1 | 6.6, 8.6 |  |  |  |
|  | 5 d |  | 12.5, 17.1 | 13.4, 14.6 | 5.0, 6.7 | 10.0, 8.5 | 5.9, 7.4 |  |  |  |
|  | 6 d |  | 11.4, 16.7 | 16.1, 21.0 | 5.9, 9.1 | 10.7, 10.9 | 6.2, 8.2 |  |  |  |
|  | 7 d |  | 7.8, 14.4 | 11.4, 18.9 | 7.0, 13.4 | 14.1, 18.4 | 3.6, 5.9 |  |  |  |
