# Supplementary figures and images for: Sustained-release lidocaine sheet for pain following tooth extraction: A randomized, single-blind, dose-response, controlled, clinical study of efficacy and safety
Source: PLoS One. 2018 Jul 2;13(7):e0200059. doi: 10.1371/journal.pone.0200059 (PMC6028143; doi:10.1371/journal.pone.0200059)

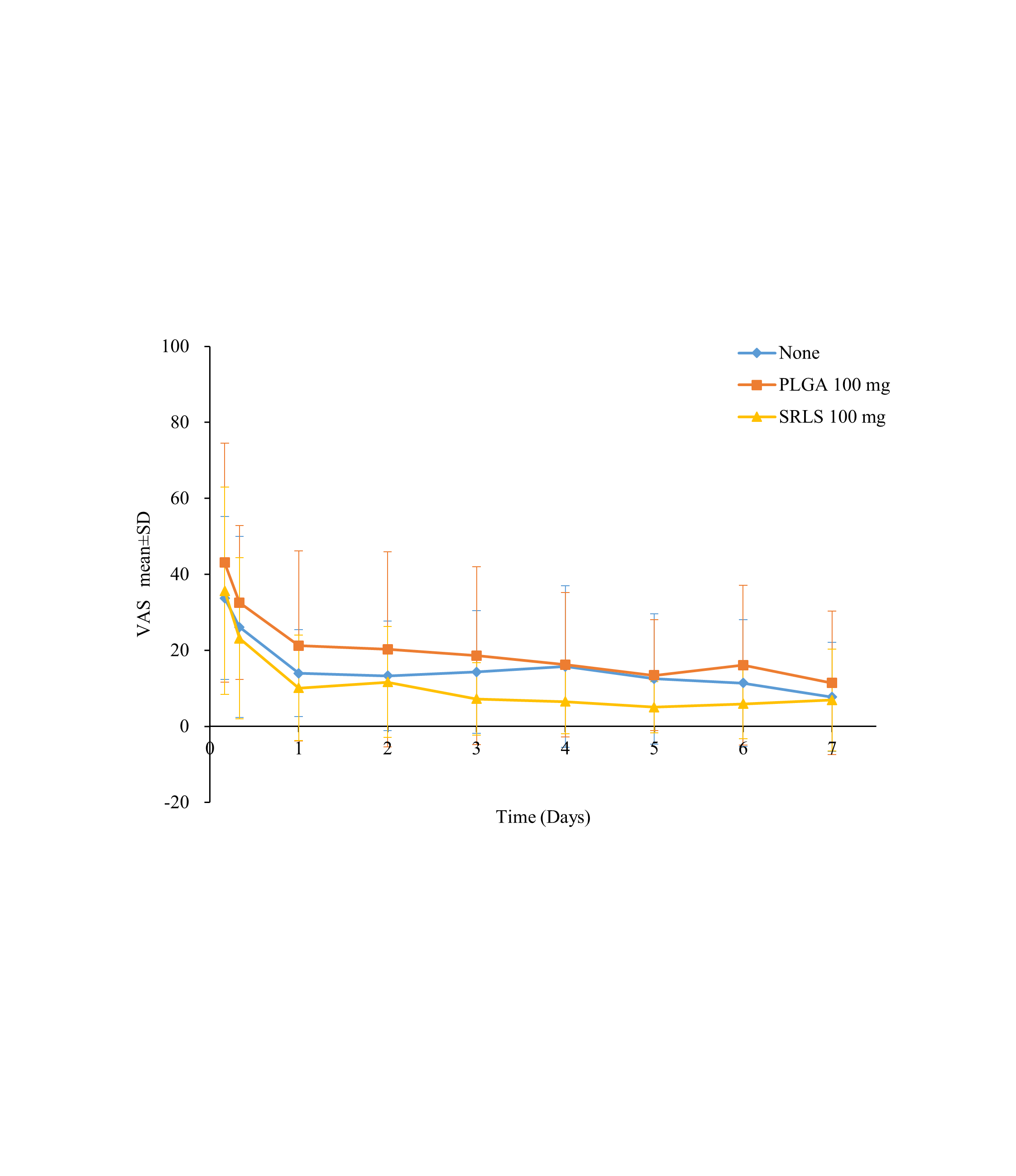

Supplement: S1 Fig — (TIF) [file pone.0200059.s006.TIF]

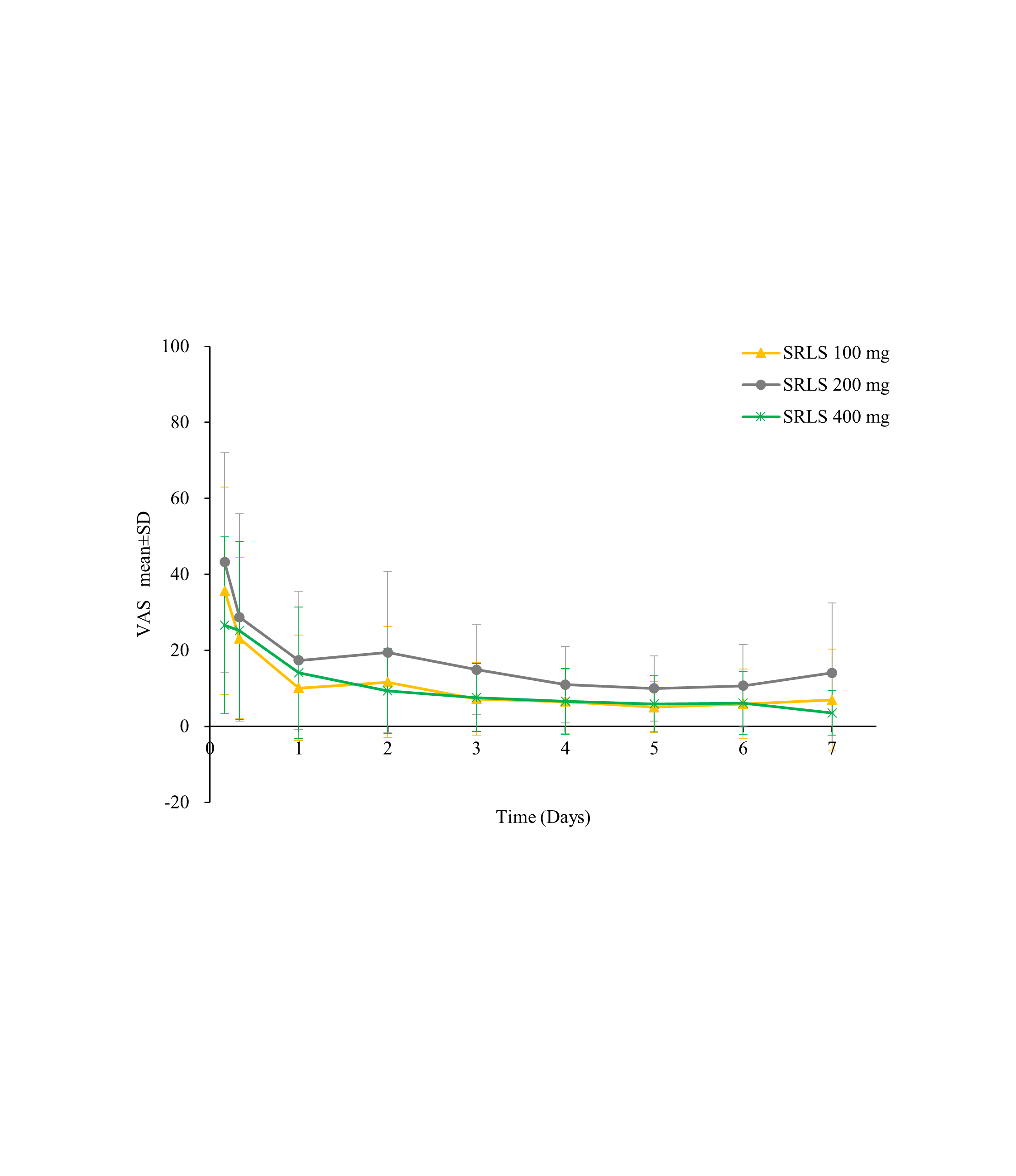

Supplement: S2 Fig — (TIF) [file pone.0200059.s007.TIF]
